# Brain and blood metabolite signatures of pathology and progression in Alzheimer disease: A targeted metabolomics study

**DOI:** 10.1371/journal.pmed.1002482

**Published:** 2018-01-25

**Authors:** Vijay R. Varma, Anup M. Oommen, Sudhir Varma, Ramon Casanova, Yang An, Ryan M. Andrews, Richard O’Brien, Olga Pletnikova, Juan C. Troncoso, Jon Toledo, Rebecca Baillie, Matthias Arnold, Gabi Kastenmueller, Kwangsik Nho, P. Murali Doraiswamy, Andrew J. Saykin, Rima Kaddurah-Daouk, Cristina Legido-Quigley, Madhav Thambisetty

**Affiliations:** 1 Clinical and Translational Neuroscience Unit, Laboratory of Behavioral Neuroscience, National Institute on Aging (NIA), National Institutes of Health (NIH), Baltimore, Maryland, United States of America; 2 Consilience Research Advisors LLP, Bengaluru, Karnataka, India; 3 HiThru Analytics, Laurel, Maryland, United States of America; 4 Department of Biostatistical Science, Wake Forest School of Medicine, Winston-Salem, North Carolina, United States of America; 5 Department of Mental Health, Johns Hopkins Bloomberg School of Public Health, Baltimore, Maryland, United States of America; 6 Department of Neurology, Duke University School of Medicine, Durham, North Carolina, United States of America; 7 Department of Pathology, Johns Hopkins University School of Medicine, Baltimore, Maryland, United States of America; 8 Department of Neurology, Houston Methodist Hospital, Houston, Texas, United States of America; 9 Rosa & Co LLC, San Carlos, California, United States of America; 10 Institute of Bioinformatics and Systems Biology, Helmholtz Zentrum München, German Research Center for Environmental Health, Neuherberg, Germany; 11 German Center for Diabetes Research (DZD), Neuherberg, Germany; 12 Department of Radiology and Imaging Sciences and the Indiana Alzheimer Disease Center, Indiana University School of Medicine, Indianapolis, Indiana, United States of America; 13 Department of Psychiatry and Behavioral Sciences, Duke University, Durham, North Carolina, United States of America; 14 Department of Medicine, Duke University, Durham, North Carolina, United States of America; 15 IPS, Faculty of Life Sciences and Medicine, King's College London, London, United Kingdom; University of Cambridge, UNITED KINGDOM

## Abstract

**Background:**

The metabolic basis of Alzheimer disease (AD) is poorly understood, and the relationships between systemic abnormalities in metabolism and AD pathogenesis are unclear. Understanding how global perturbations in metabolism are related to severity of AD neuropathology and the eventual expression of AD symptoms in at-risk individuals is critical to developing effective disease-modifying treatments. In this study, we undertook parallel metabolomics analyses in both the brain and blood to identify systemic correlates of neuropathology and their associations with prodromal and preclinical measures of AD progression.

**Methods and findings:**

Quantitative and targeted metabolomics (Biocrates AbsoluteIDQ [identification and quantification] p180) assays were performed on brain tissue samples from the autopsy cohort of the Baltimore Longitudinal Study of Aging (BLSA) (*N* = 44, mean age = 81.33, % female = 36.36) from AD (*N* = 15), control (CN; *N* = 14), and “asymptomatic Alzheimer’s disease” (ASYMAD, i.e., individuals with significant AD pathology but no cognitive impairment during life; *N* = 15) participants. Using machine-learning methods, we identified a panel of 26 metabolites from two main classes—sphingolipids and glycerophospholipids—that discriminated AD and CN samples with accuracy, sensitivity, and specificity of 83.33%, 86.67%, and 80%, respectively. We then assayed these 26 metabolites in serum samples from two well-characterized longitudinal cohorts representing prodromal (Alzheimer’s Disease Neuroimaging Initiative [ADNI], *N* = 767, mean age = 75.19, % female = 42.63) and preclinical (BLSA) (*N* = 207, mean age = 78.68, % female = 42.63) AD, in which we tested their associations with magnetic resonance imaging (MRI) measures of AD-related brain atrophy, cerebrospinal fluid (CSF) biomarkers of AD pathology, risk of conversion to incident AD, and trajectories of cognitive performance. We developed an integrated blood and brain endophenotype score that summarized the relative importance of each metabolite to severity of AD pathology and disease progression (Endophenotype Association Score in Early Alzheimer’s Disease [EASE-AD]). Finally, we mapped the main metabolite classes emerging from our analyses to key biological pathways implicated in AD pathogenesis. We found that distinct sphingolipid species including sphingomyelin (SM) with acyl residue sums C16:0, C18:1, and C16:1 (SM C16:0, SM C18:1, SM C16:1) and hydroxysphingomyelin with acyl residue sum C14:1 (SM (OH) C14:1) were consistently associated with severity of AD pathology at autopsy and AD progression across prodromal and preclinical stages. Higher log-transformed blood concentrations of all four sphingolipids in cognitively normal individuals were significantly associated with increased risk of future conversion to incident AD: SM C16:0 (hazard ratio [HR] = 4.430, 95% confidence interval [CI] = 1.703–11.520, *p* = 0.002), SM C16:1 (HR = 3.455, 95% CI = 1.516–7.873, *p* = 0.003), SM (OH) C14:1 (HR = 3.539, 95% CI = 1.373–9.122, *p* = 0.009), and SM C18:1 (HR = 2.255, 95% CI = 1.047–4.855, *p* = 0.038). The sphingolipid species identified map to several biologically relevant pathways implicated in AD, including tau phosphorylation, amyloid-β (Aβ) metabolism, calcium homeostasis, acetylcholine biosynthesis, and apoptosis. Our study has limitations: the relatively small number of brain tissue samples may have limited our power to detect significant associations, control for heterogeneity between groups, and replicate our findings in independent, autopsy-derived brain samples.

**Conclusions:**

We present a novel framework to identify biologically relevant brain and blood metabolites associated with disease pathology and progression during the prodromal and preclinical stages of AD. Our results show that perturbations in sphingolipid metabolism are consistently associated with endophenotypes across preclinical and prodromal AD, as well as with AD pathology at autopsy. Sphingolipids may be biologically relevant biomarkers for the early detection of AD, and correcting perturbations in sphingolipid metabolism may be a plausible and novel therapeutic strategy in AD.

## Abstract 2

Using quantitative and targeted metabolomics, Vijay Varma and colleagues identified metabolites for which brain tissue levels were associated with Alzheimer disease (AD) neuropathology and blood concentrations were associated with AD progression in prodromal and preclinical stages.

## Author summary

**Why was this study done?:**

- Metabolomics, which measures the biochemical products of cell processes, can be used to measure alterations in biochemical pathways related to AD.
- Several recent studies have applied metabolomics to explore potential blood biomarkers for Alzheimer disease (AD).
- Prior blood biomarker studies have not linked signals in the blood to those in the brain and have relied mainly on discriminating between AD/mild cognitive impairment (MCI) and control samples.
- These study designs ignore the long preclinical prodrome of AD and do not provide biological insights into the evolution of AD pathology in the brain and eventual development of clinical symptoms.
- Our study was designed to link alterations in metabolite signals in the brain to those in the blood, explore how those alterations were associated with distinct endophenotypes of AD, and identify the key biological pathways implicated.

**What did the research do and find?:**

- We used quantitative and targeted metabolomics assays on brain tissue samples (*N* = 44) and machine-learning methods to identify a brain metabolite signature of AD, i.e., a 26-metabolite panel that discriminated AD and control samples with accuracy, sensitivity, and specificity of 83.33%, 86.67%, and 80%, respectively.
- We then assayed the same 26 metabolites in blood from two longitudinal cohorts that represent prodromal (Alzheimer’s Disease Neuroimaging Initiative [ADNI], *N* = 767) and preclinical (Baltimore Longitudinal Study of Aging [BLSA], *N* = 207) AD and tested their associations with MRI measures, CSF biomarkers, risk of conversion to incident AD, and cognitive performance.
- We found that higher blood concentrations of sphingolipid species were consistently associated with severity of AD pathology at autopsy and AD progression across prodromal and preclinical stages.
- These metabolites map to several biologically relevant pathways in AD, including tau phosphorylation, Aβ metabolism, calcium homeostasis, acetylcholine biosynthesis, and apoptosis.

**What do these findings mean?:**

- Our study design represents a novel approach for identifying markers of disease progression in AD and potential avenues for therapeutic intervention.
- Perturbations in sphingolipid metabolism are consistently associated with preclinical and prodromal AD, as well as with AD pathology at autopsy, providing compelling evidence for their significant role in AD pathogenesis.

## Introduction

The relationships between systemic abnormalities in metabolism and the pathogenesis of Alzheimer disease (AD) are poorly understood. It is unclear how global perturbations in metabolism are related to severity of AD pathology and the eventual expression of AD symptoms in at-risk individuals. Understanding the metabolic basis of AD and its impact on disease progression during the early, preclinical, and prodromal stages is likely to provide insights into novel disease-modifying treatments for this irreversible, progressive neurodegenerative disorder.

Metabolomics, which measures the biochemical products of cell processes downstream of genomic, transcriptomic, and proteomic systems, has generated excitement because of its potential to capture snapshots of the complex and multifactorial biochemical pathways that may be altered in AD [1,2]. These include changes across multiple physiological pathways driven by the complex interactions between behavioral, genetic, and environmental risk factors. Recent studies have applied metabolomics to examine alterations in blood metabolite profiles in AD; such studies have the potential to both discover peripheral biomarkers as well as identify key metabolic pathways intrinsic to AD pathogenesis [3–7]. However, one of the key challenges in these metabolomics studies is the inability to link alterations in metabolite signals in the blood to those in the brain. It is therefore difficult to assess whether a peripheral signal associated with disease status is also reflected in the brain, where accumulation of distinct pathological features in specific regions is believed to trigger symptom onset. As is common with late-onset and gradually progressive diseases, there are many alterations in cell processes due to chronic comorbid medical conditions that may be reflected in peripheral blood metabolite concentrations. Additionally, traditional blood biomarker studies have relied mainly on the binary discrimination of established AD/mild cognitive impairment (MCI) from control (CN) samples. This study design ignores the long preclinical prodrome of AD, when brain pathology is accumulating but has not yet triggered the onset of cognitive impairment and functional decline in individuals eventually diagnosed with AD. As we have proposed previously [8], alternative study designs in biomarker analyses, in which the primary end points are well-established endophenotypes of AD pathology rather than binary discrimination of case versus control, offer the potential to identify biologically relevant blood biomarkers for AD.

**Fig 1:**
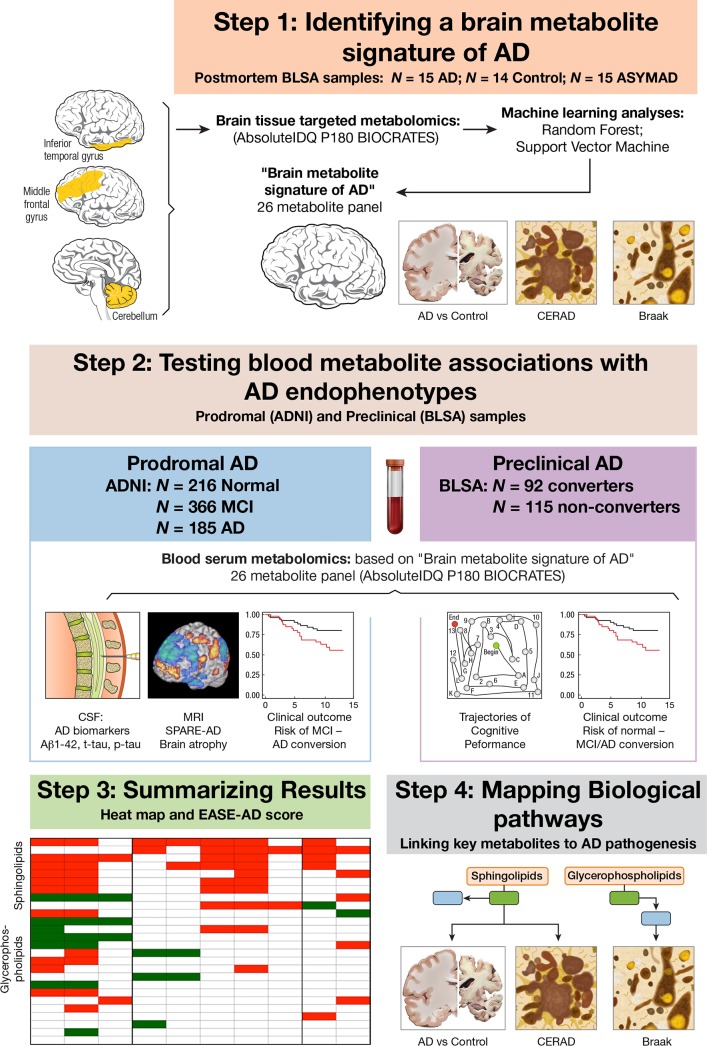
Schematic representation of study design. In Step 1, we used a quantitative and targeted metabolomics approach followed by two machine-learning methods to identify a panel of metabolites—a “brain metabolite signature of AD”—that accurately differentiated brain tissue samples from neuropathologically confirmed AD and CN subjects. In Step 2, using that same metabolite panel, we explored whether blood concentrations of metabolites in two independent samples representing prodromal AD (ADNI) and preclinical AD (BLSA) were associated with distinct clinical, cognitive, neuroimaging, and CSF endophenotypes of AD. In Step 3, we summarized results by developing an integrated blood and brain endophenotype score capturing the relative importance of specific brain and blood metabolites to severity of AD pathology and disease progression. In Step 4, we mapped the main metabolite classes (emerging from Step 3) to key biological pathways implicated in AD pathogenesis. Aβ_1–42_, amyloid beta 1–42; AD, Alzheimer disease; ADNI, Alzheimer’s Disease Neuroimaging Initiative; ASYMAD, asymptomatic Alzheimer’s disease; BLSA, Baltimore Longitudinal Study of Aging; CERAD, Consortium to Establish a Registry for Alzheimer's Disease; CN, control; CSF, cerebrospinal fluid; EASE-AD, Endophenotype Association Score in Early Alzheimer’s disease; IDQ, Identification and Quantification; MCI, mild cognitive impairment; MRI, magnetic resonance imaging; p-tau, phosphorylated tau; SPARE-AD, Spatial Patterns of Abnormality for Recognition of Early Alzheimer’s disease; t-tau, total tau.

Here, we describe a four-step approach to the discovery of brain and blood metabolites associated with pathology and progression of AD (Fig 1). (1) Identifying a brain metabolite signature of AD: in this phase of the study, we first used quantitative and targeted metabolomics to identify a panel of metabolites that accurately differentiated brain tissue samples from neuropathologically confirmed AD and CN subjects. (2) Testing blood metabolite associations with AD endophenotypes: we then tested whether serum concentrations of the same metabolites in two independent samples representing preclinical AD and prodromal AD were associated with distinct clinical, cognitive, neuroimaging, and cerebrospinal fluid (CSF) endophenotypes of AD. (3) Summarizing results: we developed an integrated blood and brain endophenotype score (Endophenotype Association Score in Early Alzheimer’s Disease [EASE-AD]) summarizing the relative importance of specific brain and blood metabolites to severity of AD pathology and disease progression. (4) Mapping biological pathways: we finally mapped the main metabolite classes emerging from these analyses to key biological pathways implicated in AD pathogenesis to understand the potential roles of these molecules and their interactions in triggering symptom onset and progression of AD.

## Methods

### Participants

The Baltimore Longitudinal Study of Aging (BLSA) is a prospective cohort study of community-dwelling participants that began in 1958 [9,10]. Detailed clinical and cognitive evaluations, including neurological, laboratory, and radiological evaluations, were conducted every 2 years. Since 2003, participants older than 80 years received yearly assessments. The autopsy subsample used in Step 1 (i.e., Identifying a brain metabolite signature) to generate the brain metabolite signature of AD included 44 participants (*N* = 15 AD; *N* = 14 CN; *N* = 15 asymptomatic Alzheimer’s disease [ASYMAD], described below). For Step 2 (i.e., Testing blood metabolite associations with AD endophenotypes), metabolomic analyses in serum samples were performed on 207 BLSA (exclusion criteria described below) participants divided into “converters” and “non-converters.” Converters were defined as participants who were cognitively normal at the initial blood draw and developed incident AD based on consensus clinical diagnosis (described below) during follow-up approximately 5 years later. These participants were age and sex matched to non-converters and defined as participants who remained cognitively normal over a similar follow-up interval. Initial serum samples were collected while both groups were cognitively normal; we therefore characterize the converters in this sample as representing “preclinical AD.” Demographic characteristics of the autopsy sample and blood study sample in the BLSA are included in Table 1. Written informed consent was obtained at each visit; the BLSA study protocol has ongoing approval with the institutional review board of the National Institute of Environmental Health Science (NIEHS), National Institutes of Health.

**Table 1:** Demographic characteristics of study samples.

**BLSA: brain (autopsy) study sample**				
	**Total Sample, *N* = 44**	**CN, *N* = 14**	**ASYMAD, *N* = 15**	**AD, *N* = 15**
Age, mean (SD)	81.33 (10.19)	80.42 (10.98)	85.19 (8.72)	78.25 (10.26)
Sex, *n* (% female)	16 (36.36)	3 (21.43)	5 (33.33)	8 (53.33)
Race, *n* (% white)	43 (97.73)	13 (92.86)	15 (100)	15 (100)
APOE e4 carrier, *n* (%)	7 (17.95)	1 (7.69)	3 (21.43)	3 (25.00)
Postmortem interval (hours), mean (SD)	14.93 (6.86)	15.82 (7.03)	14.79 (8.08)	14.4 (5.87)
**BLSA: blood study sample**				
	**Total Sample, *N* = 207**	**Non-converters, *N* = 115**	**Converters, *N* = 92**	
Age (mean, SD)	78.68 (7.23)	77.58 (7.08)	80.05 (7.23)	
Sex, *n* (% female)	107 (51.69)	55 (47.83)	52 (56.52)	
Race, *n* (% white)	172 (83.09)	89 (77.39)	83 (90.22)*	
APOE e4-carrier, *n* (%)	55 (28.65)	24 (22.64)	31 (36.05)*	
Storage time in years (mean, SD)	15.30 (6.58)	13.28 (5.98)	17.84 (6.45)*	
**ADNI: blood study sample**				
	**Total Sample, *N* = 767**	**Normal, *N* = 216**	**MCI, *N* = 366**	**AD, *N* = 185**
Age, mean (SD)	75.19 (6.82)	75.98 (5.05)	74.69 (7.35)^#^	75.26 (7.46)
Sex, *n* (% female)	327 (42.63)	105 (48.61)	132 (36.07)^#^	90 (48.65)
Race, *n* (% white)	713 (92.96)	199 (92.13)	341 (93.17)	173 (93.51)
APOE e4-carrier, *n* (%)	381 (49.67)	58 (26.85)	200 (54.64)^#^	123 (66.49)^#^
Storage time in years (mean, SD)		8.89 (0.37)	8.69 (0.42)	8.72 (0.40)

* *p* < 0.05 comparing non-converters and converters at baseline (both samples had normal cognition at baseline)

# *p* < 0.05 comparing MCI or AD to control group.

**Abbreviations:** AD, Alzheimer disease; ADNI, Alzheimer’s Disease Neuroimaging Initiative; APOE e4, apolipoprotein E epsilon 4 allele; ASYMAD, asymptomatic Alzheimer’s disease; BLSA, Baltimore Longitudinal Study of Aging; CN, control; MCI, mild cognitive impairment; SD, standard deviation.

As described below, in addition to identifying a brain metabolite signature of AD in Step 1, the BLSA sample was used in Step 2 to test associations between blood metabolite concentrations and the following AD endophenotypes: (1) differences by diagnoses (i.e., converters versus non-converters), (2) risk of conversion to incident AD, and (3) trajectories of cognitive performance prior to onset of AD symptoms.

The Alzheimer’s Disease Neuroimaging Initiative (ADNI) sample was also used in Step 2 (i.e., Testing blood metabolite associations with AD endophenotypes). ADNI is an ongoing, longitudinal study launched in 2003 as a public–private partnership, led by principal investigator Michael W. Weiner, MD. The primary goal of ADNI has been to test whether serial magnetic resonance imaging (MRI), positron emission tomography (PET), other biological markers, and clinical and neuropsychological assessments can be combined to measure the progression of mild cognitive impairment (MCI) and early AD. Details on study design, participant recruitment, study approval, and informed consent procedures have been published previously [11]. The study was approved by the institutional review boards of all of the participating institutions/study sites. The full list of participating institutions is included in S1 Table. Informed written consent was obtained from all participants at each site. Metabolomics data for ADNI samples were generated by the Alzheimer Disease Metabolomics Consortium and (ADMC) deposited to LONI. The mission of the ADMC is to create a comprehensive metabolomics database for AD. ADNI data used in the preparation of this article were also obtained from the ADNI-1 database (adni.loni.usc.edu) and included baseline blood serum metabolite concentrations (with concurrent structural MRI data) on 767 participants and concurrent CSF AD biomarker data on 403 participants. ADNI was enriched with participants with MCI and therefore represents “prodromal AD” (participants with MCI at baseline who subsequently converted back to normal cognition were excluded). Demographic characteristics of the ADNI sample are included in Table 1.

As described below, the ADNI sample was used in Step 2 to test associations between blood metabolite concentrations and the following AD endophenotypes: (1) MRI measures of AD-related brain atrophy, (2) CSF measures of AD pathology, and (3) risk of conversion to incident AD.

### Neuropathological studies: Brain tissue samples in BLSA (Step 1)

The autopsy program of the BLSA was initiated in 1986 and has been described previously [12]. The autopsy subsample is not significantly different from the BLSA cohort as a whole in terms of the rates of dementia and clinical stroke [13]. Postmortem brain examinations were performed by an experienced neuropathologist. Assessment of neuritic plaques and neurofibrillary tangles using Consortium to Establish a Registry for Alzheimer's Disease (CERAD) [14] and Braak criteria [15], respectively, have been described previously [16]. We have previously described the clinico-pathological features of BLSA participants categorized as ASYMAD after neuropathological assessment at death [17]. Briefly, these individuals had significant AD neuropathology at autopsy but were found to be cognitively intact, as assessed by longitudinal neuropsychological assessments, within 1 year prior to death.

### Determining cognitive status in BLSA and ADNI (Step 2)

In BLSA, cognitive status was considered at consensus diagnosis conferences after each assessment/visit, using established procedures described previously [18]. The consensus conferences included neurologists, neuropsychologists, and neuroimaging scientists. At each assessment, participants underwent a battery of neuropsychological testing. Clinical and neuropsychological data were reviewed at multidisciplinary consensus case conferences if they made four or more errors on the Blessed Information, Memory, and Concentration (BIMC) test, if their Clinical Dementia Rating (CDR) score was equal to or greater than 0.5, or if concerns were raised about their cognitive status by a reliable informant. In addition, all participants were evaluated by case conference on death or withdrawal. It is also important to note that longitudinal data reviewed during consensus case conferences include (besides detailed cognitive assessments) medication history, self-reported diagnoses of comorbid medical conditions, neuroimaging data, as well as laboratory evaluation for reversible causes of cognitive impairment such as serum TSH and B12 levels. The diagnoses of dementia and AD were based on the Diagnostic and Statistical Manual (DSM)-III-R [19] and the National Institute of Neurological and Communication Disorders and Stroke–Alzheimer’s Disease and Related Disorders Association (NINCDS-ADRDA) criteria, respectively [20].

For individuals diagnosed with AD, age at onset of initial symptoms of AD was estimated at consensus case conferences using longitudinal cognitive performance data as well as informant-based history.

In ADNI, dementia diagnosis was determined based on NINCDS-ADRDA criteria for probable AD. MCI participants met criteria for amnestic MCI [21], and CN participants were cognitively normal. Additional details on the ADNI protocol are available at http://www.adni-info.org.

### Blood samples in BLSA and ADNI (Step 2)

Blood serum samples were collected from BLSA participants at the NIA Clinical Research Unit in Harbor Hospital, Baltimore. Details on collection and processing have been published previously [7]. Briefly, venous blood samples were collected between 6 and 7 AM following an overnight fast. Serum samples were aliquoted into 0.5-mL volume in Nunc cryogenic tubes and stored at −80°C until further use. Samples were not subject to any freeze–thaw cycles prior to metabolomic assays. Additional details on sample selection have been published previously [7]. The average storage time of serum samples in BLSA participants was 17.84 years (SD: 6.45) in converters and 13.28 years (SD: 5.98) in non-converters (Table 1). In order to minimize potential effects of long storage times on serum sample stability and metabolite concentrations, we excluded all samples (*N* = 9 non-converters; *N* = 34 converters) with methionine sulfoxide (Met-So) concentrations greater than 5 μM (3 SD above average) [22,23]. The original sample included 250 participants; after excluding samples with high Met-So concentration, the final sample included 207 participants (*N* = 115 non-converters; *N* = 92 converters).

Details on collection and processing of ADNI blood serum samples have been published previously (http://adni.loni.usc.edu/wp-content/uploads/2010/11/BC_Plasma_Proteomics_Data_Primer.pdf). Briefly, blood was collected at 8 AM prior to CSF collection after an overnight fast, immediately placed on dry ice, and shipped on the same day to the ADNI Biomarker Core at the University of Pennsylvania for processing. The final sample included 767 participants (*N* = 216 normal; *N* = 366 MCI; *N* = 185 AD). All samples had Met-So concentrations below 5 μM and no samples were excluded.

### Brain and blood metabolomics in BLSA and ADNI (Steps 1 and 2)

Quantitative metabolomics was performed on BLSA brain and BLSA and ADNI blood samples on the Biocrates AbsoluteIDQ p180 platform. This commercially available platform allows for the quantification of amino acids, acylcarnitines, sphingomyelins (SMs), phosphatidylcholines (PCs), hexoses (h1s), and biogenic amines. Details on the assays have been published previously [24]. Briefly, the validated assay uses two different mass spectrometric methods with isotope labeled and other internal standards for absolute quantification of metabolites. The acylcarnitines, lipids, and h1s are analyzed by flow injection analysis-tandem mass spectrometry (FIA-MS/MS). The amino acids and biogenic amines are derivatized using phenylisothiocyanate and analyzed by liquid chromatography tandem-mass spectrometry (HPLC-MS/MS) using an AB SCIEX 4000 QTrap mass spectrometer (AB SCIEX, Darmstadt, Germany) with electrospray ionization. Concentration of each metabolite was measured in μM.

For brain tissue metabolomics, regions were selected a priori in the middle frontal gyrus (MFG), inferior temporal gyrus (ITG), and cerebellum (CBL). The MFG and ITG were sampled to represent brain regions vulnerable to amyloid β and tau deposition, respectively; the CBL was sampled to represent a brain region resistant to classical AD pathology. A sterile 4-mm-diameter tissue punch was extracted from the cortical surface of the brain tissue regions, which were stored at −80°C. To extract metabolites, samples were homogenized using Precellys with ethanol phosphate buffer; samples were then centrifuged, and the supernatant was used for analysis. Metabolite concentrations in brain tissue samples indicated as less than the limit of detection (LOD) were imputed as the highest value below the LOD. This method removed all differences below the LOD but still allowed machine-learning classifiers to pick up any differences in metabolite concentrations between those above the LOD and those below.

For blood metabolomics, in BLSA, converter and non-converter samples were randomly divided into 6 batches. Each batch was processed in separate runs with technicians blinded to diagnostic status. Additional data processing and checking steps, including reproducibility and testing for equality of coefficient of variance across metabolites, has been described in detail previously [25]. BLSA serum samples indicated as less than LOD were not imputed due to minimal missingness; 25/26 metabolites had 0 < LOD values. ADNI data processing has been described in detail previously and included imputing values indicated as less than the LOD as the metabolite LOD/2, as determined by the ADMC [25]. Metabolite concentrations from participants with duplicate measurements were averaged in all analyses.

Batch effects were controlled for using a set of CN samples. Standardized quality control (QC) material, i.e., commercially available pooled human plasma spiked with a defined set of metabolites, was used across all batches to control and adjust for batch effects by applying MetIDQ software-implemented normalization procedures.

### Cognitive assessments in BLSA (Step 2)

Cognitive performance was analyzed from assessments administered to BLSA participants every two years. Memory was assessed using the California Verbal Learning Test (CVLT), including learning (total recall over 5 learning trials), immediate free recall, and long delay free recall. Attention was assessed using the Trails Making Test Part A and the WAIS-R Digits Forward test. Executive function was measured using the Trails Making Test Part B and the WAIS-R Digit Backward test. Language was measured using letter fluency and semantic fluency tests. Visuo-spatial ability was measured using the Clock Drawing Test and the Card Rotation Test.

### Structural MRI measures in ADNI (Step 2)

MRI protocol, including scanner specifications, image acquisition, and image processing, are described in detail at www.adni-info.org. Briefly, protocol specifications included T1 weighted MR images, including sagittal volumetric 3D MPRAGE with 1.25 × 1.25-mm in-plane spatial resolution, 1.2-mm thick sagittal slices, 8° flip angle, and target TR of about 8.9 mm and TE of about 3.9 ms [26]. We utilized the Spatial Patterns of Abnormality for Recognition of Early Alzheimer’s disease (SPARE-AD) index [27] as a neuroimaging measure of “AD-like” brain atrophy patterns [28]; this measure was calculated for baseline visits of ADNI-1 participants.

### CSF measures of AD pathology in ADNI (Step 2)

Participants underwent lumbar puncture in the morning following overnight fasting and blood draws. Samples were immediately placed on dry ice and shipped to the ADNI Biomarker Core for processing. Total tau (t-tau), phosphorylated tau (p-tau), and amyloid beta 1–42 (Aβ_1–42_) were measured using the multiplex xMAP Luminex platform (Luminex Corp, Austin, TX) with Innogenetics (INNO-BIA AlzBio3, Ghent, Belgium) immunoassay kit-based reagents. See http://adni.loni.usc.edu/wp-content/uploads/2012/01/2011Dec28-Biomarkers-Consortium-Data-Primer-FINAL1.pdf for additional details on sample collection and processing, including reproducibility and data quality checks. CSF samples were available in 395 participants (*N* = 109 normal; *N* = 186 MCI; *N* = 100 AD).

### Statistical analysis

The first two stages of the analytic plan used for BLSA, including Step 1, Identifying a brain metabolite signature of AD, and Step 2, Testing blood metabolite associations with AD endophenotypes, were developed conceptually in May 2016 prior to any data analyses. There were no subsequent data-driven alterations to this conceptual analytic plan; final data visualization for Step 3, Summarizing results, was based on various iterations during analyses. The inclusion of ADNI data occurred in fall 2016 after our data request was approved by the ADMC. Step 4, Mapping biological pathways, occurred after we identified the principal classes of metabolites emerging from Steps 1–3. Sensitivity analyses testing blood metabolite associations with AD endophenotypes in BLSA (indicated below in Step 2) were conducted at the request of reviewers.

#### Step 1: Identifying a brain metabolite signature of AD

Absolute brain tissue concentrations of 187 targeted metabolites were generated using the quantitative metabolomic methods described previously on the MFG, ITG, and CBL in the autopsy subsample of the BLSA. We used two machine-learning methods, support vector machine (SVM) and random forest (RF), to generate average values of classification accuracy, sensitivity, and specificity for discriminating between postmortem AD and CN samples in each of the three brain regions examined. Both machine-learning methods are based on different principles (described below) and were used in combination to avoid bias towards a particular methodology when selecting relevant metabolites. The prediction models use the selected metabolites in combination with each other, effectively modeling interactions between them. The primary aim of the machine-learning analyses was to discriminate between AD and CN samples, and therefore the ASYMAD group was not included in defining the brain metabolite signature of AD.

Briefly, SVM is a classification method that attempts to find a separating surface between two classes with maximum margin [29]. If there is no separating surface in the original feature space, SVM uses a kernel to implicitly map the features into a higher dimensional space, in which a separating surface can be found. The performance of an SVM classifier on test data has rigorous theoretical bounds [29], and it is possible to limit the “complexity” of the prediction model to match the amount of data available. It has been shown that complex classifier models that have many parameters that can be tuned to match the training data perform poorly on test data due to overfitting. The restriction on the complexity of the SVM classifier has been found to generalize well to test data, particularly when the number of features (*p*) is much greater than the number of samples (*n*) [30].

Since its inception in 2001, RF has become popular in the machine-learning and bioinformatics communities [31]. RF is one of the so-called ensemble methods for classification, because a set of classifiers (instead of one) is generated and each one casts a vote for the predicted label of a given instance provided to the model. Each classifier is a tree built using the classification and regression trees (CART) methodology [32]. RF often requires little tuning of the parameters. RF is nonlinear, multivariate, and can deal with high-dimensional data, even in small sample size situations. RF contains built-in metrics of variable importance, which allow evaluating the relative relevance of each variable in a RF model. In the present report, we used the permutation index of variable importance, which quantifies decreases in accuracy of the estimated RF model due to random permutation of a given variable. Additional details on using SVM and RF methodologies to discriminate between diseased and non-diseased individuals in AD have been published previously [33]. To estimate metrics of performance (accuracy, sensitivity, specificity) we used leave-one-out (LOO) cross validation.

The SVM and RF methods generated a ranked list of the top metabolites that contributed to discriminating between AD and CN samples. The ranking for SVM was based on the number of cross-validation iterations that each metabolite was selected in (i.e., higher numbers indicating higher rank), while that for RF was based on the mean decrease in accuracy when a particular metabolite was excluded from the prediction model. Because both methods rely on different analytic principles and differences in feature selection, we expected that the top metabolites from each method would not necessarily be identical; using both in combination therefore avoided bias when defining the brain metabolite signature.

The ITG samples had the highest accuracy and sensitivity/specificity in discriminating AD from CN samples. The top 20 ranked metabolites from each machine-learning classifier (SVM and RF) in this region (ITG) were therefore used to define the brain metabolite signature of AD.

#### Step 1: Identifying a brain metabolite signature of AD: Differences by group and associations with AD pathology

We next explored differences in concentration of each brain tissue metabolite across 3 groups—AD, CN, and ASYMAD—in the ITG. Importantly, these analyses included the ASYMAD group, which was not utilized in the development of the brain metabolite signature of AD through the machine-learning analyses. Concentrations of brain tissue metabolites were natural log transformed. Proportional odds ordinal logistic models, a generalization of the Wilcoxon and Kruskal-Wallis test, were used to test for differences across groups (i.e., AD, ASYMAD, CN) using brain tissue metabolite concentration as the outcome, group as the nominal predictor, and age at death and sex as covariates. We then explored associations between brain tissue metabolite concentrations and severity of AD pathology, specifically CERAD and Braak scores, again using all three groups, including ASYMAD. Spearman’s rank correlation tests, adjusting for age at death and sex, were used to test these associations.

#### Step 2: Testing blood metabolite associations with AD endophenotypes: Risk of conversion to incident AD in cognitively normal older adults (BLSA)

In the BLSA sample, we explored whether the natural log-transformed blood concentration of each metabolite identified in the brain metabolite signature of AD was associated with risk of conversion to incident AD. We used Cox regression models, a class of survival models, to explore whether the initial concentration of each metabolite (i.e., while all participants were cognitively normal) was associated with the time to onset of conversion to AD. We included the covariates, age at initial blood draw and sex, in the model; individuals who remained normal (non-converters) at follow-up were censored at their last visit. Hazard ratios (HRs) indicate the relative increase in the hazard rate associated with 1 log-unit increase in concentration of the log-transformed metabolite. An HR greater than 1.0 indicates that higher log-transformed concentration of the metabolite is associated with increased risk, while an HR less than 1.0 indicates that lower concentration of the log-transformed metabolite is associated with increased risk.

#### Step 2: Testing blood metabolite associations with AD endophenotypes: Associations with cognitive performance (BLSA)

Using the metabolites identified in the brain metabolite signature of AD, we next explored whether the natural log-transformed blood concentration of each metabolite was associated with longitudinal trajectories of cognitive performance in cognitively normal individuals who developed incident AD. We first generated domain-specific composite scores within the following domains: memory, attention, executive function, language, and visuospatial ability using methods described previously [24]. These methods are also described in detail in S1 Appendix. Briefly, composite scores were calculated by summing and averaging the standardized scores from multiple tests within each cognitive domain. Linear mixed effects regression models were used to test whether the concentration of each metabolite was associated with longitudinal changes in domain-specific cognitive performance in cognitively normal individuals converting to incident AD. All models included the following predictors: natural log-transformed metabolite concentration, age at initial blood draw, sex, time (in days between follow-up visit and baseline; baseline indicated as time = 0), and the two-way interaction of each predictor with time. The main predictor of interest was the interaction of metabolite concentration with time, which indicates an increase or decrease in the annualized rate of change in domain-specific cognitive performance associated with an increase in metabolite concentration. As our main goal in these analyses was to examine associations between blood metabolite concentrations and progression of AD during the early preclinical stage of disease, we excluded all cognitive performance data after the onset of AD symptoms.

#### Step 2: Testing blood metabolite associations with AD endophenotypes in BLSA (sensitivity analyses)

Due to differences in serum sample storage time among converters and non-converters in the BLSA cohort and a greater number of converter samples excluded by the Met-So cutoff, we performed additional sensitivity analyses within a subsample of converters and non-converters. After excluding all samples with Met-So concentration >5 μM, we matched converter to non-converter serum samples on the duration of sample storage at −80°C within a range of ±2 years. This produced a matched sample of 74 converters (storage time: 16.15 [SD: 5.83]) and 74 non-converters (storage time: 15.89 [SD: 5.90]). In these sensitivity analyses, we tested whether significant associations observed in the original dataset between serum metabolites and (i) risk of conversion to incident AD and (ii) cognitive performance remained significant after matching on storage time.

#### Step 2: Testing blood metabolite associations with AD endophenotypes: Associations with AD-like brain atrophy patterns and CSF biomarkers of AD pathology (ADNI)

Using the metabolites identified in the brain metabolite signature of AD, we next explored cross-sectional associations between natural log-transformed blood metabolite concentrations and the SPARE-AD index, a measure of AD-related brain atrophy derived from MRI scans [27]. We used multivariate linear regression, including the following predictors: natural log-transformed baseline metabolite concentration, baseline age, and sex; the outcome was the SPARE-AD index (higher scores represent more “AD-like” brain atrophy patterns).

In the ADNI sample, we additionally examined cross-sectional associations between natural log-transformed baseline blood metabolite concentrations and natural log-transformed CSF t-tau, p-tau, and Aβ_1–42_ concentrations. Similar to the model for the SPARE-AD analysis, multivariate linear regression models included the following predictors: natural log-transformed baseline metabolite concentration, baseline age, and sex; the outcomes were natural log-transformed CSF t-tau, p-tau, and Aβ_1–42_ concentrations.

#### Step 2: Testing blood metabolite associations with AD endophenotypes: Associations with risk of conversion to incident AD (ADNI)

In the ADNI sample, we explored whether the natural log-transformed blood concentration of each metabolite identified in the brain metabolite signature of AD was associated with risk of conversion from MCI to incident AD. Similar to survival models used in BLSA, Cox regression models were used to explore whether initial metabolite concentrations in MCI participants were associated with the time to onset of conversion to AD. We included covariates, age at baseline blood draw, and sex in the model; individuals who remained MCI at follow-up were censored at their last visit. Similar to survival models used in BLSA, the HR indicates the relative increase in the hazard rate associated with 1 log-unit increase in concentration of the log-transformed metabolite. An HR greater than 1.0 indicates that a higher log-transformed concentration of the metabolite is associated with increased risk, while an HR less than 1.0 indicates that a lower concentration of the log-transformed metabolite is associated with increased risk.

#### Step 3: Summarizing results: Calculating the EASE-AD score

In order to visually summarize results from all analyses of metabolites comprising the brain metabolite signature of AD and to explore whether metabolites clustered by class in their associations with distinct AD-related endophenotypes, we generated a heat map indicating statistically significant associations between AD metabolites (y-axis) and the specific brain and blood endophenotypes (x-axis) described above. Significant associations (*p* < 0.05) are highlighted in red or green indicating that increased or decreased metabolite concentration, respectively, is associated with the various AD-related endophenotypes. Nonsignificant associations are indicated in gray. The 26-metabolite panel (brain metabolite signature of AD) was determined specifically based on metabolite rankings from the machine-learning classifiers following rigorous cross validation and thus represent a priori hypotheses in subsequent analyses. Additionally, these secondary, exploratory analyses were all focused on testing the associations of these metabolites with distinct measures of AD progression in order to identify consistent trends across two independent cohorts [34]. For these reasons, we elected not to use a *p*-value correction in the secondary analyses.

In order to enhance ease of interpretation of the summary heat map, we collapsed all longitudinal domain-specific cognitive performance tests into one category indicating significant longitudinal associations in any domain. We included the following brain endophenotype categories: (1) Differences in brain metabolite concentrations in the ITG by diagnosis (i.e., AD, CN, and ASYMAD) and correlations of metabolite concentrations in the ITG with (2) CERAD and (3) Braak scores. We included the following blood metabolite versus preclinical AD endophenotype categories (BLSA): associations of blood metabolite concentrations with (4) risk of progression from normal to incident AD and (5) longitudinal trajectories of cognitive performance. We included the following blood metabolite versus prodromal AD endophenotype categories (ADNI): associations of blood metabolite concentrations with (6) AD-like brain atrophy patterns on MRI (i.e., SPARE-AD score), (7) CSF Aβ_1–42_, (8) CSF t-tau, (9) CSF p-tau, and (10) risk of progression from MCI to incident AD. We then calculated a summary EASE-AD score indicating the number of significant associations for each AD metabolite with brain and blood endophenotypes (max score: 10). This score is included as the last column in the heat map; visualized metabolites are sorted based on this score in order to explore clustering of metabolite species within main classes.

We present detailed results for representative metabolites that showed significant associations with multiple AD phenotypes. Detailed results for all 26 AD metabolites are included in Supporting information tables (S3 Table–S11 Table).

#### Step 4: Mapping biological pathways

In order to interpret our results within the biological context of the metabolic pathways implicated, we mapped the principal metabolite classes emerging from our analyses to their known primary biosynthetic and catabolic pathways as well as their known interactions through various enzymatically regulated intermediary reactions (“Metabolic pathway”). We also mapped the main metabolite classes implicated to key signaling mechanisms related to AD pathogenesis (“Signaling pathway”).

## Results

### Participants: Demographic characteristics

The demographic characteristics of BLSA participants in the autopsy cohort whose brain tissue samples were used in the metabolomics assays are included in Table 1. The mean age at death in the autopsy sample was 81.33 years (SD: 10.19), and the mean interval between last evaluation and death (postmortem interval) was 14.93 h (SD: 6.86). Participants in the three groups—CN, ASYMAD, and AD—did not significantly vary by age at death, sex, or postmortem interval. The demographic characteristics of BLSA participants who provided blood data are included in Table 1. Participants were aged 78.47 years (SD: 6.96) at initial blood draw and 51.69% were female. Converter and non-converter groups did not vary by age or sex. Serum samples in the converter group were, on average, stored for four years longer than non-converter samples (17.84 years [SD: 6.45] versus 13.28 years [SD: 5.98]; *p* < 0.05).

The demographic characteristics of ADNI participants are included in Table 1. Participants were aged 75.19 years (SD: 6.82) at baseline, and 42.63% were female. MCI participants were significantly younger (74.69 years [SD: 7.35]) and had fewer females (36.07%). Samples did not vary by storage time.

#### Step 1: Identifying a brain metabolite signature of AD

Accuracy, sensitivity, and specificity of the machine-learning classifiers in discriminating between AD and CN samples for the MFG, ITG, and CBL brain regions are included in Table 2. The SVM algorithm identified a panel of metabolites that discriminated samples in the ITG with an accuracy of 83.33% and a sensitivity/specificity of 86.67%/80.00%. The RF algorithm derived metabolites that discriminated samples in the ITG with an accuracy of 70.00% and a sensitivity/specificity of 66.70%/73.30%. The performance metrics of metabolite concentrations in discriminating samples in the MFG and CBL were comparatively lower.

**Table 2:** Machine-learning methods to discriminate between AD and CN samples.

RF		SVM	
ITG			
Accuracy	70.00%	Accuracy	83.33%
Sensitivity	66.70%	Sensitivity	86.67%
Specificity	73.30%	Specificity	80.00%
MFG			
Accuracy	58.60%	Accuracy	31.03%
Sensitivity	60.00%	Sensitivity	46.67%
Specificity	57.10%	Specificity	14.29%
CBL			
Accuracy	34.60%	Accuracy	38.46%
Sensitivity	53.70%	Sensitivity	60.00%
Specificity	9.10%	Specificity	9.09%

**Abbreviations:** AD, Alzheimer disease; CBL, cerebellum; CN, control; ITG, inferior temporal gyrus; MFG, middle frontal gyrus; RF, random forest; SVM, support vector machine.

Based on the performance of our machine-learning algorithms in the ITG, we chose the top 20 ranked metabolites from the SVM and RF algorithms from this region to define the brain metabolite signature of AD. Out of 187 metabolites assayed, these top metabolites thus contributed the most to discriminating between pathology-confirmed AD cases and CN in the ITG. We found that 13 “consensus” metabolites were shared in the top 20 of both SVM and RF algorithms, and 7 metabolites in each were unique to each algorithm (i.e., a total of 27 metabolites—13 consensus metabolites and 14 unique metabolites). We excluded one metabolite, h1, that was not available in the ADNI dataset from subsequent analyses. Table 3 presents the top 27 metabolites, including the 13 consensus metabolites; full ranked lists from both SVM and RF algorithms from the ITG are included in S2 Table.

**Table 3:** Top metabolites based on SVM and RF algorithms in the ITG.

Abbreviation	Full Metabolite Name
**Amino Acid**	
Arg	Arginine
**Acylcarnitine**	
C3	Propionylcarnitine
**Glycerophospholipids**	
lysoPC a C17:0	Lysophosphatidylcholine with acyl residue C17:0
lysoPC a C18:0*	Lysophosphatidylcholine with acyl residue C18:0
PC aa C38:4	Phosphatidylcholine with diacyl residue sum C38:4
PC aa C40:4*	Phosphatidylcholine with diacyl residue sum C40:4
PC aa C40:5	Phosphatidylcholine with diacyl residue sum C40:5
PC aa C40:6*	Phosphatidylcholine with diacyl residue sum C40:6
PC ae C34:0*	Phosphatidylcholine with acyl-alkyl residue sum C34:0
PC ae C34:2	Phosphatidylcholine with acyl-alkyl residue sum C34:2
PC ae C36:0*	Phosphatidylcholine with acyl-alkyl residue sum C36:0
PC ae C36:3	Phosphatidylcholine with acyl-alkyl residue sum C36:3
PC ae C36:4	Phosphatidylcholine with acyl-alkyl residue sum C36:4
PC ae C40:1	Phosphatidylcholine with acyl-alkyl residue sum C40:1
PC ae C42:3*	Phosphatidylcholine with acyl-alkyl residue sum C42:3
**Biogenic Amines**	
Serotonin	Serotonin
Spermidine*	Spermidine
**Sphingolipids**	
SM C16:0	Sphingomyelin with acyl residue sum C16:0
SM C16:1*	Sphingomyelin with acyl residue sum C16:1
SM C18:1	Sphingomyelin with acyl residue sum C18:1
SM C24:1*	Sphingomyelin with acyl residue sum C24:1
SM C26:1*	Sphingomyelin with acyl residue sum C26:1
SM (OH) C14:1	Hydroxysphingomyelin with acyl residue sum C14:1
SM (OH) C22:1*	Hydroxysphingomyelin with acyl residue sum C22:1
SM (OH) C22:2*	Hydroxysphingomyelin with acyl residue sum C22:2
SM (OH) C24:1*	Hydroxysphingomyelin with acyl residue sum C24:1

* Indicates consensus metabolites common to both SVM and RF algorithms.

**Abbreviations:** ITG, inferior temporal gyrus; RF, random forest; SVM, support vector machine.

#### Step 1: Identifying a brain metabolite signature of AD: Differences by group and associations with AD pathology

**Fig 2:**
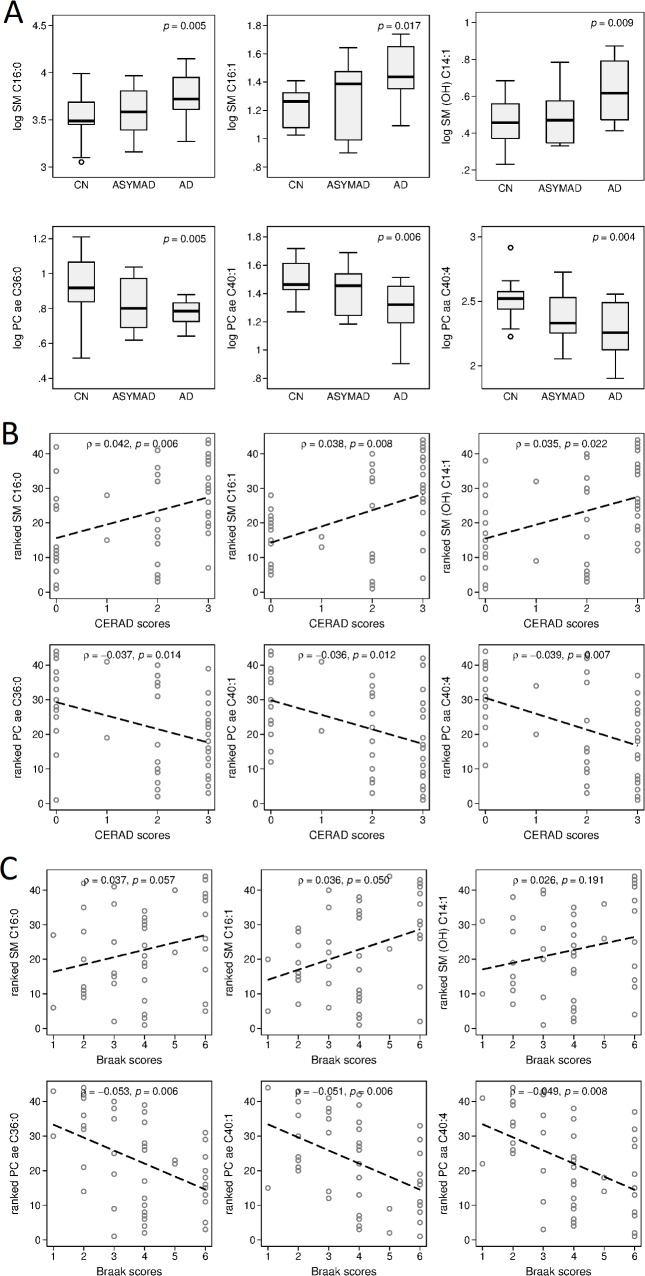
Associations between brain tissue metabolite concentration and clinical groups, CERAD scores, and Braak scores. Please note that vertical axes scales differ across graphs in panels A and B. (**A**) Group differences and global *p*-values for significance across clinical groups for brain tissue concentration of three representative sphingolipids and three representative glycerophospholipids in the ITG. (**B**) ρs and *p*-values showing associations between three representative sphingolipids and three representative glycerophospholipids and severity of neuritic plaque burden (CERAD scores). (**C**) ρs and *p*-values showing associations between three representative sphingolipids and three representative glycerophospholipids and severity of neurofibrillary pathology (Braak scores). ρ, correlation coefficient; AD, Alzheimer disease; ASYMAD, asymptomatic Alzheimer’s disease; CERAD, Consortium to Establish a Registry for Alzheimer's Disease; CN, control; ITG, inferior temporal gyrus; OH, hydroxy; PC, phosphatidylcholine; SM, sphingomyelin.

A total of 16 metabolites showed brain tissue concentrations in the ITG that differed significantly across clinical groups, i.e., CN, ASYMAD, and AD. The majority of these were sphingolipids (8 out of 16) and glycerophospholipids (5 out of 16). The AD group generally showed the highest or lowest metabolite concentrations, while the CN group showed the opposite. The ASYMAD group generally showed intermediate metabolite concentrations between the AD and CN samples. In Fig 2A, we show group differences and global *p*-values for significance across clinical groups for brain tissue concentrations of 3 representative sphingolipids: SM C16:0 (*p* = 0.005), SM C16:1 (*p* = 0.017), and SM (OH) C14:1 (*p* = 0.009) and three representative glycerophospholipids: PC ae C36:0 (*p* = 0.005), PC ae C40:1 (*p* = 0.006), and PC aa C40:4 (*p* = 0.004). A summary of results across all metabolites is included in S3 Table.

Brain tissue concentrations of 17 metabolites were significantly associated with severity of neuritic plaque burden, as reflected in the CERAD scores. The majority (a total of 14 out of 17) of these were sphingolipids (7 out of 17) and glycerophospholipids (7 out of 17). Brain tissue concentrations of five metabolites were significantly associated with neurofibrillary pathology, as assessed by Braak scores: three glycerophospholipids, one sphingolipid, and the amino acid, arginine. Increased concentration of sphingolipids was consistently associated with greater CERAD and Braak scores. In Fig 2B and 2C, we show correlation coefficient (ρ) and *p*-value results from adjusted Spearman rank correlation tests (CERAD and Braak, respectively) for three representative sphingolipids: SM C16:0 (CERAD: ρ = 0.042, 95% CI = 0.0130–0.070, *p* = 0.006; Braak: ρ = 0.037, 95% CI = −0.001–0.076, *p* = 0.057), SM C16:1 (CERAD: ρ = 0.038, 95% CI = 0.011–0.065, *p* = 0.008; Braak: ρ = 0.036, 95% CI = 0.000–0.073, *p* = 0.050), SM (OH) C14:1 (CERAD: ρ = 0.035, 95% CI = 0.005–0.064, *p* = 0.022; Braak: ρ = 0.025, 95% CI = −0.013–0.065, *p* = 0.191); and three representative glycerophospholipids: PC ae C36:0 (CERAD: ρ = −0.037, 95% CI = −0.066–−0.008, *p* = 0.014; Braak: ρ = −0.053, 95% CI = −0.089–−0.016, *p* = 0.006), PC ae C40:1 (CERAD: ρ = −0.036, 95% CI = −0.064–−0.008, *p* = 0.012; Braak: ρ = −0.051, 95% CI = −0.086–−0.016, *p* = 0.006), and PC aa C40:4 (CERAD: ρ = −0.039, 95% CI = −0.067–−0.011, *p* = 0.007; Braak: ρ = −0.049, 95% CI = −0.085–−0.014, *p* = 0.008). A summary of results across all metabolites is included in S4 Table.

#### Step 2: Testing blood metabolite associations with AD endophenotypes: Risk of conversion to incident AD in cognitively normal older adults (BLSA)

The mean interval between initial blood sampling to the onset of AD (for converters) or follow-up (for non-converters) was 4.27 years (SD = 1.33 years). Higher blood concentrations of four sphingolipids were associated with a significantly greater risk of future conversion to incident AD in cognitively normal older individuals. These included SM C16:0 (HR = 4.430, 95% CI = 1.704–11.520, *p* = 0.002), SM C16:1 (HR = 3.455, 95% CI = 1.516–7.873, *p* = 0.003), SM (OH) C14:1 (HR = 3.539, 95% CI = 1.373–9.122, *p* = 0.009) and SM C18:1 (HR = 2.255, 95% CI = 1.047–4.855, *p* = 0.038). Lower and higher baseline blood concentrations of two glycerophospholipids, PC aa 38:4 (HR = 0.253, 95% CI = 0.102–0.630, *p* = 0.003) and PC ae C34:2 (HR = 3.055, 95% CI = 1.211–7.705, *p* = 0.018), respectively, were also associated with a significantly greater risk of conversion to incident AD. A summary of results across all metabolites is included in S5 Table.

All metabolites significantly associated with greater risk of future conversion to incident AD (i.e., 6 out of 6 metabolites) remained significant in sensitivity analyses conducted in the subsample matched on storage time. A summary of the results from the sensitivity analyses is included in S6 Table.

#### Step 2: Exploring associations in blood with AD endophenotypes: Associations with cognitive performance (BLSA)

We found that initial blood concentrations of six metabolites predicted longitudinal trajectories of domain-specific cognitive performance prior to the onset of cognitive impairment in cognitively normal individuals converting to incident AD. Higher baseline blood concentrations of the sphingolipids were broadly associated with greater declines in cognition. Specifically, higher baseline blood concentration of SM C18:1 and SM C26:1 were predictive of greater declines in attention (SM C18:1: β = −0.172, 95% CI = −0.306–0.038, *p* = 0.012) and language (β = −0.533, 95% CI = −1.061–−0.005, *p* = 0.050), respectively. Similarly, higher blood concentrations of the glycerophospholipids PC aa C40:6 and PC ae C40:1 were also predictive of greater declines in attention (PC aa C40:6: β = −0.122, 95% CI = −0.215–−0.030, *p* = 0.010) and language (PC ae C40:1: β = −0.251, 95% CI = −1.061–−0.005, *p* = 0.041). Higher blood concentration of lysoPC a C18:0 were predictive of greater declines in language (lysoPC a C18:0: β = −0.1530, 95% CI = −0.266–−0.0330, *p* = 0.012) and lower blood concentration of the polyamine, spermidine, was associated with greater declines in visuospatial ability (β = 1.220, 95% CI = 0.094–2.347, *p* = 0.034), and higher and lower blood concentration of arginine were associated with greater declines in language (β = −0.142, 95% CI = −0.270–−0.013, *p* = 0.031) and visuospatial ability (β = 0.198, 95% CI = 0.007–0.390, *p* = 0.043), respectively. Due to conflicting direction of associations between serum arginine concentrations and cognitive performance, we arbitrarily chose to indicate the association between arginine and language only, in the summary heat map. Significant associations are summarized in S7 Table.

The majority of metabolites (i.e., 6 out of 8) remained significant in sensitivity analyses conducted in the subsample matched on storage time. A summary of the results from the sensitivity analyses is included in S8 Table.

### Step 2: Testing blood metabolite associations with AD endophenotypes: Associations with AD-like brain atrophy patterns and CSF biomarkers of AD pathology (ADNI)

**Fig 3:**
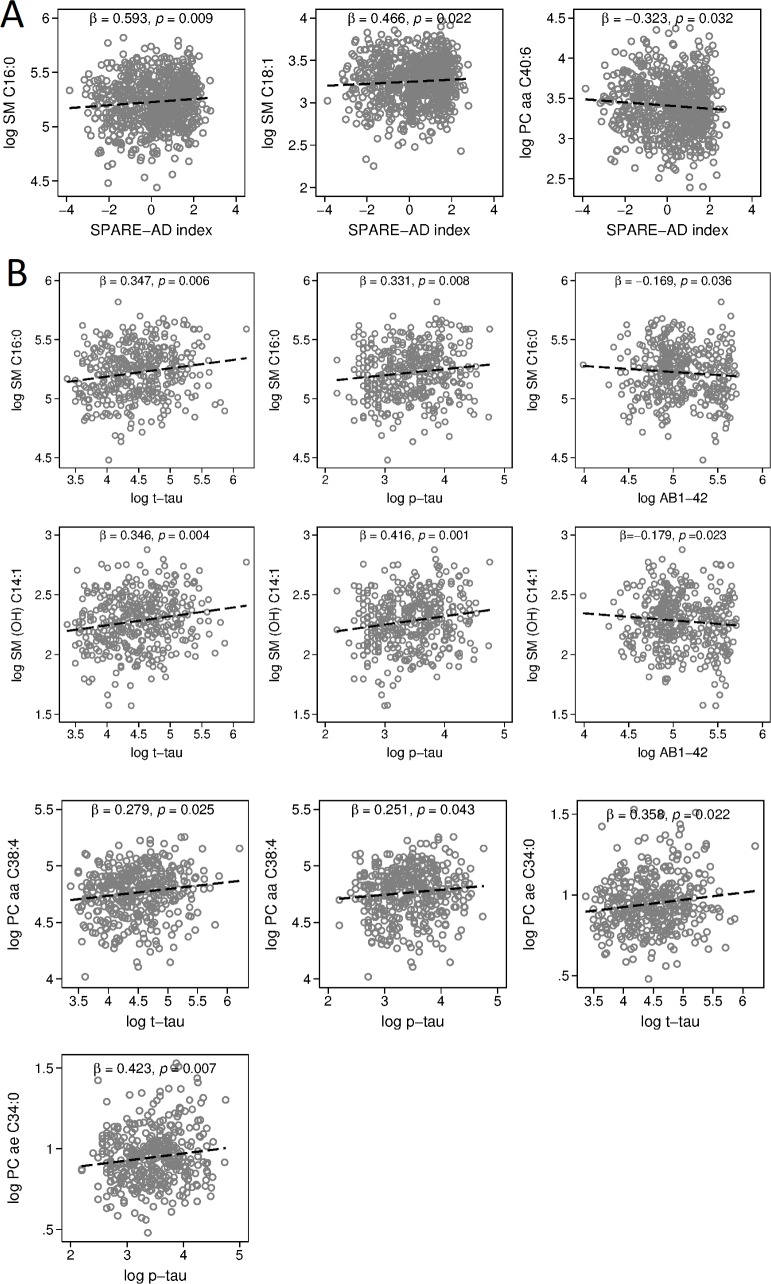
Associations between blood metabolite concentration and SPARE-AD index, CSF concentrations of Aβ_1–42_, t-tau, and p-tau. Please note that vertical axes scales differ across graphs in panels A and B. (**A**) ρs and *p*-values showing associations between representative metabolites and AD-like patterns of brain atrophy on MRI scans (SPARE-AD index). (**B**) ρs and *p*-values showing associations between representative metabolites and CSF markers of AD: Aβ_1–42_, t-tau, and p-tau. ρ, correlation coefficient; Aβ_1–42_, amyloid beta 1–42; AD, Alzheimer disease; CSF, cerebrospinal fluid; MRI, magnetic resonance imaging; OH, hydroxyl; p-tau, phosphorylated tau; PC, phosphatidylcholine; SM, sphingomyelin; SPARE-AD, Spatial Patterns of Abnormality for Recognition of Early Alzheimer’s disease; t-tau, total tau.

Higher blood concentrations of sphingolipids were broadly associated with greater AD-like brain atrophy patterns and more AD-like CSF levels of pathology. Specifically, two sphingolipids, SM C16:0 (β = 0.593, 95% CI = 0.147–1.040, *p* = 0.009) and SM C18:1 (β = 0.466, 95% CI = 0.0687–0.863, *p* = 0.022), were associated with more AD-like patterns of brain atrophy on MRI scans measured by the SPARE-AD index. Lower blood concentration of the glycerophospholipid, PC aa C40:6 (β = −0.323, 95% CI = −0.617–−0.029, *p* = 0.032), was associated with a more “AD-like” pattern of brain atrophy. Fig 3A shows all significant cross-sectional associations between blood concentrations of metabolites described above and the SPARE-AD index. A summary of results across all metabolites is included in S9 Table.

Higher blood concentrations of eight metabolites were associated with greater CSF levels of t-tau, while higher concentrations of 10 metabolites were associated with greater CSF levels of p-tau. All significant associations were among either sphingolipids (t-tau: 6 out of 8; p-tau: 8 out of 10) or glycerophospholipids (t-tau: 2 out of 8; p-tau: 2 out of 10). Higher blood concentrations of two of these sphingolipids (SM C16:0 and SM [OH] C14:1) were also associated with lower CSF levels of Aβ_1–42_. Lower blood concentrations of C3 and serotonin were also associated with lower CSF levels of Aβ_1–42_. Fig 3B shows associations between blood concentrations of SM C16:0 (t-tau: β = 0.347, 95% CI = 0.103–0.592, *p* = 0.006; p-tau: β = 0.331, 95% CI = 0.086–0.575, *p* = 0.008; Aβ_1–42_: β = −0.169, 95% CI = −0.328–−0.011, *p* = 0.036) and SM [OH] C14:1 (t-tau: β = 0.346, 95% CI = 0.109–0.583, *p* = 0.004; p-tau: β = 0.416, 95% CI = 0.179–0.653, *p* = 0.001; Aβ_1–42_: β = −0.179, 95% CI = −0.333–−0.025, *p* = 0.023), with all three CSF biomarkers. We additionally show associations between blood concentrations of PC aa C38:4 (t-tau: β = 0.279, 95% CI = 0.035–0.522, *p* = 0.025; p-tau: β = 0.251, 95% CI = 0.008–0.494, *p* = 0.043) and PC ae C34:0 (t-tau: β = 0.358, 95% CI = 0.053–0.663, *p* = 0.022; p-tau: β = 0.423, 95% CI = 0.118–0.728, *p* = 0.007) and CSF t-tau and p-tau. A summary of significant results across all metabolites is included in S10 Table.

### Step 2: Testing blood metabolite associations with AD endophenotypes: Associations with risk of conversion to incident AD (ADNI)

The mean interval between baseline blood sampling to the onset of AD or follow-up (for individuals who remained MCI) was 2.97 years (SD = 2.33 years). Higher blood concentration of one sphingolipid, SM C18:1 (HR = 2.351, 95% CI = 1.268–4.360, *p* = 0.007), was associated with a significantly greater risk of conversion to incident AD among individuals with MCI. This sphingolipid was also associated with greater risk of conversion to incident AD among cognitively normal individuals (described above in BLSA results). Higher blood concentration of one glycerophospholipid, PC aa 38:4 (HR = 2.375, 95% CI = 1.189–4.745, *p* = 0.014), was also associated with a significantly greater risk of conversion to incident AD. A summary of results across all metabolites is included in S11 Table.

### Step 3: Summarizing results: Calculating the EASE-AD score

**Fig 4:**
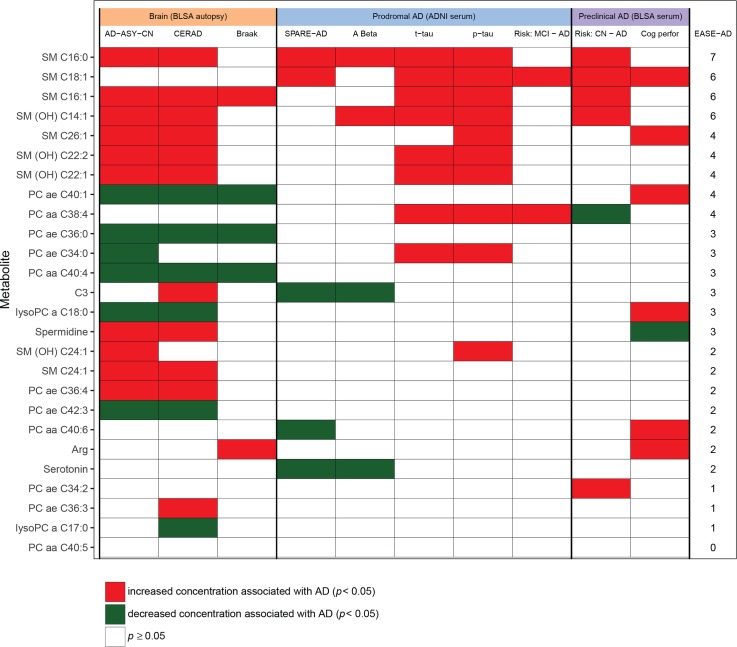
Heat map summarizing associations between metabolites and AD endophenotypes. Meanings of column headings: AD-ASY-CN, association between brain tissue metabolite concentration and clinical diagnosis of AD; CERAD, association between brain tissue metabolite concentration and plaques measured by CERAD score; Braak, association between brain tissue metabolite concentration and neurofibrillary tangle burden measured by Braak score; SPARE-AD, association between blood tissue metabolite concentration in ADNI and SPARE-AD score; A Beta, association between blood tissue metabolite concentration in ADNI and CSF Aβ_1–42_; t-tau, association between blood tissue metabolite concentration in ADNI and CSF (t-tau); p-tau, association between blood tissue metabolite concentration in ADNI and CSF (p-tau); Cog perfor, association between blood tissue metabolite concentration and cognitive performance prior to AD onset; EASE-AD, sum of significant associations across AD-related endophenotypes. ADNI Cox: association between blood tissue metabolite concentration and risk of incident AD in ADNI among MCI individuals. BLSA Cox: association between blood tissue metabolite concentration and risk of incident AD/MCI in BLSA among cognitively normal individuals. Aβ_1–42_, amyloid beta 1–42; AD, Alzheimer disease; ADNI, Alzheimer’s Disease Neuroimaging Initiative; ASY, asymptomatic Alzheimer’s disease; BLSA, Baltimore Longitudinal Study of Aging; CERAD, Consortium to Establish a Registry for Alzheimer's Disease; CN, control; CSF, cerebrospinal fluid; EASE-AD, Endophenotype Association Score in Early Alzheimer’s disease; MCI, mild cognitive impairment; OH, hydroxyl; p-tau, phosphorylated tau; PC, phosphatidylcholine; SM, sphingomyelin; SPARE-AD, Spatial Pattern of Abnormality for Recognition of Early Alzheimer’s disease; t-tau, total tau.

Fig 4 shows the heat map summarizing statistically significant associations between the metabolites and brain and blood-specific AD endophenotypes. Metabolites are ranked (in decreasing order) based on their EASE-AD score. *P*-values from each cell in Fig 4 are included in S12 Table.

### Step 4: Mapping biological pathways: Exploring metabolite interactions and their impact on AD pathology

**Fig 5:**
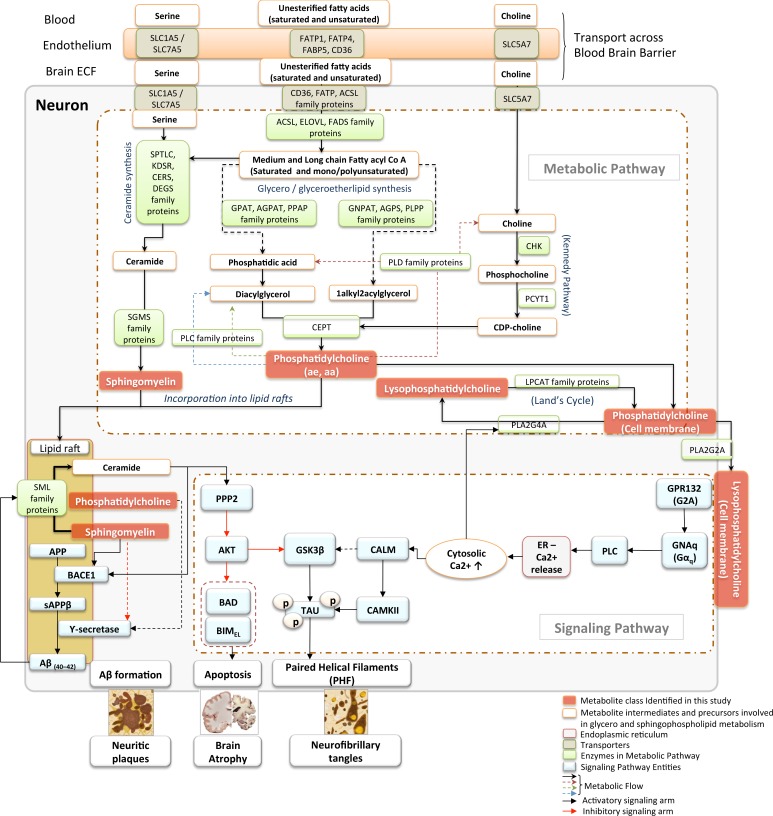
Metabolic pathways and signaling cascades involving glycerophospholipids and sphingolipids: relevance to AD pathogenesis. Schematic articulation of the core metabolic and signaling pathways in neurons, highlighting links between glycerophospholipid and sphingolipid classes of lipid species identified in the current study to be associated with the severity of AD pathology in the brain. Nutrient transporters (SLC5A7, SLC1A5, CD36, FATPs) present both at the BBB as well as the neuronal cell membrane mediate the uptake of amino acids, long chain fatty acids, and vitamin precursors into neurons necessary for the de novo synthesis of glycerophospholipid and SM lipid species [35,36]. The “metabolic pathway” section of the diagram represents the core metabolic pathways involved in the synthesis and recycling of glycerophospholipid and sphingolipid species. The “signaling pathway” section connects these lipid species to the core representative signaling cascades implicated in mediating multiple aspects of AD pathology in the brain, such as formation of neuritic plaques, neurofibrillary tangles, and AD-like brain atrophy. In a condition-dependent manner, incoming free fatty acids are incorporated into glycerolipids or ceramides in the endoplasmic reticulum. Similarly, LCFAs are processed in peroxisomal organelles to generate ether lipids. Coupling with the Kennedy pathway, glycerolipids and ether lipids are converted to either aa or ae PC species [37]. PCs are metabolized by the phospholipase or SML enzymes to recycle back phosphatidic acid or DAG or to generate SM, respectively. These lipid species are critical in the formation of lipid rafts, which represent essential structural and functional domains for maintaining neuronal function [38]. In AD, remodeling of lipid rafts, especially with enhanced activity of SMLs, results in an increased ceramide to SM ratio, which facilitates Aβ production by posttranslational stabilization of BACE1 enzyme. This leads to further generation of oligomeric Aβ due to a feed forward regulatory loop between Aβ and the SML enzymes [39]. Similarly, PC with saturated and unsaturated long-chain fatty acyl groups positively influence activity of the Ƴ-secretase enzyme by modulating cell membrane thickness and the lipid microenvironment of the enzyme [40]. Meanwhile, generation of lysophosphatidylcholine from membrane PC by both cytosolic PLA2G4A in Land’s cycle [41] as well as the secretory soluble PLA2G2A can lead to dysregulation of intracellular calcium signaling in a G-protein receptor (GPR132, G2A) coupled manner. Dysregulated Ca2+ signaling can result in enhanced activity of CAMKII, which, in coordination with the ceramide–PP2A–GSK3β pathway, results in tau hyperphosphorylation, leading to the generation of PHF and enhanced neurofibrillary tangle formation [42]. Furthermore, altered ceramide signaling by down-regulation of AKT kinase activity via PP2A can trigger neuronal apoptosis by augmenting activity of the pro-apoptotic proteins, BAD and BIM_EL_. aa, diacyl; Aβ, amyloid-β; AD, Alzheimer disease; ae, acyl-alkyl; AKT, protein kinase B; BACE1, β-secretase; BAD, BCL2 associated agonist of cell death; BBB, blood-brain barrier; BIM_EL_, BCL2 interacting mediator of cell death-extra long; CAMKII, calmodulin kinase; CD36, CD36 molecule; DAG, diacylglycerol; ECF, extracellular fluid; ER, endoplasmic reticulum FATP, fatty acid transport protein; LCFA, long-chain fatty acid; LysoPC, lysophosphatidylcholine; PC, phosphatidylcholine; PHF, paired helical filaments; PLA2G2A, phospholipase A2 group IIA; PLA2G4A, phospholipase A2 Group IVA; PP2A, protein phosphatase; SLC1A5, solute carrier family 1 member 5; SLC5A7, solute carrier family 5 member 7; SM, sphingomyelin; SML, sphingomyelinase.

Fig 5 summarizes the main biosynthetic and catabolic reactions (“Metabolic pathway”) of the major metabolite classes and their interactions as well as their roles in signaling cascades (“signaling pathway”) relevant to AD pathogenesis and evolution of the principal pathological features of the disease.

#### Gene symbol and name

**SLC5A7**, solute carrier family 5 member 7; **SLC1A5**, solute carrier family 1 member 5; **SLC7A5**, solute carrier family 7 member 5; **FATP1**, fatty acid transport protein-1; **FATP4**, fatty acid transport protein-4; **FABP5**, fatty acid binding protein 5; **CD36**, CD36 molecule; **SPTLC**, serine palmitoyltransferase long chain base; **KDSR**, 3-ketodihydrosphingosine reductase; **CERS**, ceramide synthase; **DEGS**, delta 4-desaturase, sphingolipid; **SGMS**, sphingomyelin synthase; **SML**, sphingomyelinase; **ACSL**, acyl-CoA synthetase long-chain family member; **ELOVL**, elongation of very long chain fatty acids; **FADS**, fatty acid desaturase; **GNPAT**, glyceronephosphate O-acyltransferase; **AGPS**, alkylglycerone phosphate synthase; **PLPP**, phospholipid phosphatase; **GPAT**, Glycerol-3-phosphate acyltransferases; **AGPAT**, 1-acylglycerol-3-phosphate O-acyltransferase; **PPAP**, phospholipid phosphatase; **PLD,** phospholipase D; **CHK**, choline kinase; **PCYT1**, phosphate cytidylyltransferase 1, choline; **CEPT**, choline/ethanolamine phosphotransferase; **PLA2G2A**, phospholipase A2 group II A; **PLA2G4A**, phospholipase A2 group IV A; **LPCAT**, lysophosphatidylcholine acyltransferase; **GPR132 (G2A)**, G protein-coupled receptor 132; **GNAq**, G protein subunit alpha q; **PLC**, phospholipase C; **CALM**, calmodulin; **CAMKII**, calmodulin kinase II; **GSK3B**, glycogen synthase kinase 3 beta; **TAU**, microtubule-associated protein tau; **PPP2**, serine/threonine protein phosphatase II; **AKT**, protein kinase B (PKB); **BAD**, BCL2 associated agonist of cell death; **BIM**_**EL**_, BCL2 interacting mediator of cell death (BIM)-extra long; Aβ_40–42_, amyloid beta (40–42); **BACE1**, beta-secretase 1; **APP**, amyloid protein precursor; **sAPPβ**, soluble amyloid protein precursor beta.

## Discussion

To the best of our knowledge, this is the first study to apply quantitative and targeted metabolomic analyses of both brain and blood tissue to identify metabolites associated with the severity of AD pathology as well as measures of AD progression. Our results indicate that distinct metabolites belonging to the sphingolipid and glycerophospholipid classes are related to the severity of AD pathology in the brain and that their concentrations in blood are associated with preclinical disease progression. Furthermore, we were able to identify these specific metabolites through a data-driven process that first used machine-learning methods to generate an AD-specific brain metabolite signature, and then clustered these metabolites based on the EASE-AD summary score representing cumulative associations of each metabolite, with outcome measures related to AD pathology and progression.

### Sphingolipids and AD

This process identified sphingolipids as a class of metabolites that are consistently associated with preclinical and prodromal AD, as well as with AD pathology at autopsy. Additionally, for all sphingolipid species—across all endophenotypes in brain, prodromal, and preclinical blood samples—increased concentration was associated with a more “AD-like” phenotype. Our results add substantially to a growing body of literature suggesting that perturbations in sphingolipid metabolism are related to key aspects of AD pathogenesis [43,44]. SMs are a subclass of sphingolipids that are enriched in the central nervous system as important constituents of lipid rafts [38] and play a critical role in neuronal cell signaling [45,46]. In the brain, sphingolipids mediate a diverse array of biological functions that are relevant to critical molecular mechanisms in AD, including amyloidogeneic processing of the amyloid precursor protein (APP) within SM-rich lipid rafts [47] and regulation of hippocampal neuronal excitability [48]. While previous studies in postmortem human brain tissue have demonstrated altered levels of total SM content in AD relative to CN [49,50], few have quantified absolute concentrations of distinct SM species within brain regions differentially vulnerable to AD pathology. Our findings are broadly consistent with those of Chan and colleagues, who demonstrated higher levels of the SM species SM d18:1/22:1 and d18:1/26:1 in the prefrontal and entorhinal cortices of AD patients, relative to CN [51].

Most previous studies reporting on altered blood sphingolipid levels in AD have used an untargeted lipidomics approach (e.g., [52,53]). Some recent studies have used the p180 targeted metabolomics platform to assay absolute concentrations of metabolites associated with AD. An important distinction in the design of these previous studies and our current report is in our use of a brain-derived AD metabolite signature to guide focused analyses of these metabolites in blood as well as a comprehensive exploration of their associations within both preclinical and prodromal AD samples. Two studies [25,54] have recently reported on p180 metabolite data within blood samples in the ADNI and the Atherosclerosis Risk in Communities (ARIC) cohorts. While there is minimal overlap between these results and our current report, it is striking to note that two sphingolipids we observe to be increased in the temporal cortex of AD patients and identified in our brain metabolite signature of AD (SM C16:0 and SM [OH] C14:1) were associated with brain atrophy, cognitive decline, and risk of conversion from MCI to AD in ADNI [25]. Similarly, blood concentrations of SM C16:0 and SM C26:1 were also associated with a diagnosis of MCI and dementia, respectively, in the predominantly African-American ARIC cohort [54].

Our findings that blood concentration of sphingolipids represented in the brain metabolite signature of AD are also associated with progression during preclinical and prodromal AD suggest that these are biologically relevant, early signals of disease progression. Equally importantly, correcting perturbations in sphingolipid metabolism may represent a plausible novel strategy for therapeutic intervention in AD. In this context, the emerging roles of sphingosine 1-phosphate (S1P)-metabolizing enzymes and S1P analogs in ameliorating Aβ-induced neuroinflammation in AD [55,56] are especially promising.

### Glycerophospholipids and AD

The second major class of metabolites we observed to be related to measures of AD pathology were glycerophospholipids (i.e., PCs and lysophosphatidylcholines [LysoPCs]). The majority of associations between these metabolites were in the brain tissue samples: generally, lower concentrations of glycerophospholipids were associated with greater severity of both amyloid and neurofibrillary pathology; associations between glycerophospholipids and preclinical and prodromal AD endophenotypes were sparse. In previous studies using untargeted and semiquantitative metabolomics, we demonstrated that AD patients show lower plasma concentrations of distinct phosphatidylcholines (PC aa C36:5, PC aa C38:6, and PC aa C40:6), relative to CN [57]. We recently extended these findings to show that reduced plasma concentration of these phosphatidylcholines is also related to lower levels of cognitive performance in non-demented older individuals and reflects resting state cerebral blood flow (rCBF), a marker of neuronal activity, in several brain regions related to higher order cognitive processing [58]. Taken together, these prior findings and our current results add further evidence for a role of altered phosphatidylcholine metabolism in AD pathogenesis.

### Network biology: Metabolic pathway alterations in AD

In order to develop an integrated understanding of central–peripheral lipid metabolite fluxes as well as interactions between the major metabolite classes observed in this study, we applied a network biology approach. Fig 5 summarizes these networks, based on prior knowledge of transport mechanisms related to these metabolites and their precursors as well as their known biosynthetic pathways and catabolic fates. Long-chain fatty acid (LCFA) precursors for glycerophospholipid and sphingolipid biosynthesis are transported both across the blood-brain barrier (BBB) and through plasma membranes within the brain through protein-mediated active transport by fatty acid transport proteins (FATPs), long-chain acyl-CoA synthetases (ACSLs), fatty acid binding proteins (FABPs), and the fatty acid transporter (FAT)/CD36 [35,36]. In the context of neurodegenerative diseases in general and AD in particular, transport of the omega-3 (ω-3) polyunsaturated fatty acid (PUFA), docosahexaenoic acid (DHA; 22:6n-3), into the brain is especially important [59,60]. In a recent untargeted lipidomic analysis in brain tissue samples from the BLSA, we showed that dysregulation of fatty acid metabolism is associated with severity of AD pathology [24]. Fig 5 also shows key enzymatically regulated steps in the biosynthesis of phosphatidylcholines through the Kennedy pathway [37] and their reversible conversion to LysoPCs through Land’s cycle [41]. The transfer of phosphocholine headgroups to ceramides by the enzyme phosphatidylcholine transferase (sphingomyelin synthase [SGMS]) is a key intermediary step in sphingolipid biosynthesis [43] and is a potentially critical link between glycerophospholipid and sphingolipid metabolism observed in our current report.

By performing our initial discovery analyses in brain tissue samples at autopsy and subsequent validation in preclinical (i.e., BLSA) and prodromal (i.e., ADNI) serum samples, we were able to ask whether metabolic changes associated with markers of AD neuropathology in established disease are similar to blood metabolite changes in early AD pathogenesis. Broadly, our results indicate that there are shared pathways between metabolite changes in brain and blood, with the prodromal serum samples (i.e., ADNI) sharing more metabolites with brain samples than the preclinical (i.e., BLSA) serum samples (see Fig 4). A plausible explanation for these findings is that blood metabolite changes associated with later stages of AD progression prior to symptom onset are more similar to metabolic correlates of AD pathology in established disease. In independent analyses, we have also used the BLSA and ADNI serum samples as discovery datasets to ask whether principal metabolites associated with preclinical and prodromal AD-related endophenotypes in blood are also represented among the brain metabolites (i.e., in “established disease”) reported in the current study. Among serum metabolites previously shown to be associated with AD progression in BLSA, we find that propionylcarnitine (C3) concentration in serum discriminates between converter and non-converter samples [7], and its concentration in the ITG is related to the severity of neuritic plaque pathology in our current study (Fig 4). Among serum metabolites previously shown to be associated with AD endophenotypes in ADNI, we find that SMC (OH) C14:1 and SM 16:0 concentrations in serum are associated with CSF Aβ, concentration, brain atrophy, cognitive decline, and risk of MCI progression [25], and their concentrations in the ITG (i.e., our current report) are both related to the severity of neuritic plaque pathology and differ across the three groups studied (Fig 4). Taken together, while these findings suggest that there are metabolic pathways common to both AD-related neuropathology and blood-related disease progression, there are also those that are specific to disease stage and tissue compartment. Establishing the relative importance of common and distinct metabolic pathways across tissue types and disease stages will require subsequent studies in larger datasets.

### Limitations

Our study has limitations. First, the relatively small number of brain tissue samples in our primary analyses may have limited our power to detect significant associations with other metabolites assayed and precluded the use of a discovery and validation dataset. The small number reflects the challenges of assembling brain tissue samples from well-characterized, longitudinally followed participants who also undergo detailed neuropathological assessment at death; future studies in larger brain samples are needed to validate our findings. Second, while the Biocrates AbsoluteIDQ platform is a standardized platform for multiplexed quantitative analysis of 187 different metabolites, these metabolites represent only a small proportion of the brain and blood metabolomes. Future analyses will expand our study framework across additional classes of metabolites. Third, it must be noted that we based our primary analyses on metabolites associated with AD pathology in brain tissue samples. In future studies, it would be important to perform similar analyses in cognitively normal individuals using primary outcomes derived from neuroimaging/CSF-based measures of early AD pathology in prodromal/preclinical AD. Fourth, testing of pre-analytical variables in the BLSA serum samples indicated a potential selection bias: converter samples were subject to longer storage time at −80°C, compared to non-converter samples (approximately 17 years versus 13 years, respectively; Table 1). Additionally, the converter group compared to the non-converter group had more samples above the cutoff values for Met-So concentration used as an indicator of sample quality. Therefore, we performed sensitivity analyses within a subsample of converters and non-converters matched on storage time. In these sensitivity analyses, we confirmed that 10 of the 12 metabolites associated with AD-related outcomes in the BLSA serum samples (Fig 4) remained significant. We therefore interpret these sensitivity analyses to suggest that our observed results on serum metabolite concentrations in BLSA are not driven primarily by group differences in sample storage time or quality. Finally, it is important to note that the BLSA is a predominantly Caucasian sample of highly educated and relatively healthy older individuals. Our findings therefore merit confirmation in other cohorts with higher prevalence of cardiovascular and cerebrovascular disease.

### Conclusions

In summary, we have applied quantitative and targeted metabolomics to identify a panel of sphingolipids, the concentrations of which, in brain tissue, are associated with severity of AD neuropathology and, in blood, with measures of progression during preclinical and prodromal AD. We propose that perturbations in sphingolipid metabolism may be integral to the evolution of AD neuropathology as well as to the eventual expression of AD symptoms in cognitively normal older individuals. Our study design, which takes a machine-learning and data-driven approach to identify blood metabolites associated with AD progression and explores how those metabolites are integrated within biologically relevant pathways, suggests a novel framework for identifying markers for early detection and potential avenues for effective therapeutic intervention in AD.

## Supporting information

S1 STROBE Checklist Checklist of items that should be included in reports of observational studies. (DOCX)

S1 Appendix Description of methods used to generate cognitive domain-specific composite scores. (DOCX)

S1 Table ADNI participating institutions/study sites. ADNI, Alzheimer’s Disease Neuroimaging Initiative.
(DOCX)

S2 Table Full ranking from SVM and RF algorithms from the ITG. ITG, inferior temporal gyrus; RF, random forest; SVM, support vector machine.
(DOCX)

S3 Table Brain endophenotype associations: Differences by group. (DOCX)

S4 Table Brain endophenotype associations: Associations with AD pathology. AD, Alzheimer disease.
(DOCX)

S5 Table Blood endophenotype associations: Risk of progression to incident AD in cognitively normal older individuals (BLSA). AD, Alzheimer disease; BLSA, Baltimore Longitudinal Study of Aging.
(DOCX)

S6 Table Sensitivity analyses in subsample matched on storage time. Blood endophenotype associations: risk of progression to incident AD in cognitively normal older individuals (BLSA). AD, Alzheimer disease; BLSA, Baltimore Longitudinal Study of Aging.
(DOCX)

S7 Table Blood endophenotype associations: Cognitive performance (BLSA). BLSA, Baltimore Longitudinal Study of Aging.
(DOCX)

S8 Table Sensitivity analyses in subsample matched on storage time: Blood endophenotype associations: Cognitive performance (BLSA). BLSA, Baltimore Longitudinal Study of Aging.
(DOCX)

S9 Table Blood endophenotype associations: AD-like brain atrophy patterns and CSF biomarkers of AD pathology (ADNI). AD, Alzheimer disease; ADNI, Alzheimer’s Disease Neuroimaging Initiative; CSF, cerebrospinal fluid.
(DOCX)

S10 Table Blood endophenotype associations: AD-like brain atrophy patterns and CSF biomarkers of AD pathology (ADNI). AD, Alzheimer disease; ADNI, Alzheimer’s Disease Neuroimaging Initiative; CSF, cerebrospinal fluid.
(DOCX)

S11 Table Blood endophenotype associations: Risk of progression to incident AD in MCI individuals (ADNI). AD, Alzheimer disease; ADNI, Alzheimer’s Disease Neuroimaging Initiative; MCI, mild cognitive impairment.
(DOCX)

S12 Table *P*-values for all brain- and blood-specific AD endophenotypes included in Fig 4. AD, Alzheimer disease.
(DOCX)

## Abbreviations

ρ: correlation coefficient
ω-3: omega-3
aa: diacyl
Aβ_1–42_: amyloid beta 1–42
Aβ: amyloid-β
ACSL: acyl-CoA synthetase
AD: Alzheimer disease
ADMC: Alzheimer Disease Metabolomics Consortium
ADNI: Alzheimer’s Disease Neuroimaging Initiative
ae: acyl-alkyl
AKT: protein kinase B
APOE e4: apolipoprotein E epsilon 4 allele
APP: amyloid precursor protein
ARIC: Atherosclerosis Risk in Communities
ASYMAD: asymptomatic Alzheimer’s disease
BACE1: β-secretase
BAD: BCL2 associated agonist of cell death
BBB: blood-brain barrier
BIMC: Blessed Information, Memory, and Concentration
BLSA: Baltimore Longitudinal Study of Aging
CAMKII: calmodulin kinase
CART: classification and regression trees methodology
CBL: cerebellum
CDR: Clinical Dementia Rating
CERAD: Consortium to Establish a Registry for Alzheimer's Disease
CI: confidence interval
CN: control
CSF: cerebrospinal fluid
CVLT: California Verbal Learning Test
C3: propionylcarnitine
DAG: diacylglycerol
DHA: docosahexaenoic acid
DSM: Diagnostic and Statistical Manual
EASE-AD: Endophenotype Association Score in Early Alzheimer’s Disease
ECF: extracellular fluid
ER: endoplasmic reticulum
FABP: fatty acid binding protein
FAT: fatty acid transporter
FATP: fatty acid transport protein
FIA-MS/MS: flow injection analysis-tandem mass spectrometry
HPLC-MS/MS: liquid chromatography tandem-mass spectrometry
HR: hazard ratio
h1: hexose
IDQ: identification and quantification
ITG: inferior temporal gyrus
LCFA: long-chain fatty acid
LOD: limit of detection
LOO: leave-one-out
LysoPC: lysophosphatidylcholine
MCI: mild cognitive impairment
Met-So: methionine sulfoxide
MFG: middle frontal gyrus
MRI: magnetic resonance imaging
n: number of samples
NIEHS: National Institute of Environmental Health Science
NINCDS-ADRDA: National Institute of Neurological and Communication Disorders and Stroke–Alzheimer’s Disease and Related Disorders Association
p: number of features
p-tau: phosphorylated tau
PC: phosphatidylcholine
PET: positron emission tomography
PHF: paired helical filaments
PLA2G2A: phospholipase A2 group IIA
PLA2G4A: phospholipase A2 Group IVA
PP2A: protein phosphatase
PUFA: polyunsaturated fatty acid
QC: quality control
rCBF: resting state cerebral blood flow
RF: random forest
SD: standard deviation
SGMS: sphingomyelin synthase
SLC1A5: solute carrier family 1 member 5
SLC5A7: solute carrier family 5 member 7
SM: sphingomyelin
SML: sphingomyelinase
SPARE-AD: Spatial Patterns of Abnormality for Recognition of Early Alzheimer’s disease
SVM: support vector machine
S1P: sphingosine 1-phosphate
t-tau: total tau
