# Supplementary material for: Brain and blood metabolite signatures of pathology and progression in Alzheimer disease: A targeted metabolomics study
Source: PLoS Med. 2018 Jan 25;15(1):e1002482. doi: 10.1371/journal.pmed.1002482 (PMC5784884; doi:10.1371/journal.pmed.1002482)
Supplement: S1 STROBE Checklist — (DOCX) [file pmed.1002482.s001.docx]

**STROBE Statement—checklist of items that should be included in reports of observational studies**

|  | Item No. | Recommendation | Page  No. | Relevant text from manuscript |
| --- | --- | --- | --- | --- |
| **Title and abstract** | 1 | (*a*) Indicate the study’s design with a commonly used term in the title or the abstract | Abstract: Methods and Findings | …Baltimore Longitudinal Study of Aging |
|  |  | (*b*) Provide in the abstract an informative and balanced summary of what was done and what was found | Abstract: Methods and Findings | Quantitative and targeted metabolomics….We found that distinct… |
| Introduction | | | |  |
| Background/rationale | 2 | Explain the scientific background and rationale for the investigation being reported | Introduction: paragraph 1 & 2 | The relationships between systemic abnormalities in metabolism and the pathogenesis of Alzheimer’s disease (AD) are poorly understood…  Metabolomics, which measures the biochemical products of cell processes… |
| Objectives | 3 | State specific objectives, including any prespecified hypotheses | Introduction: paragraph 3 | Here we describe a four-step approach to the discovery of brain and blood metabolites… |
| Methods | | | |  |
| Study design | 4 | Present key elements of study design early in the paper | Introduction: paragraph 3 | See Figure 1 (Schematic representation of Study Design) |
| Setting | 5 | Describe the setting, locations, and relevant dates, including periods of recruitment, exposure, follow-up, and data collection | Methods: Participants | The Baltimore Longitudinal Study of Aging (BLSA) is a prospective cohort study of community-dwelling participants that began in 1958…  ADNI is an ongoing, longitudinal study launched in 2003 as a public-private partnership… |
| Participants | 6 | (*a*) *Cohort study*—Give the eligibility criteria, and the sources and methods of selection of participants. Describe methods of follow-up  *Case-control study*—Give the eligibility criteria, and the sources and methods of case ascertainment and control selection. Give the rationale for the choice of cases and controls  *Cross-sectional study*—Give the eligibility criteria, and the sources and methods of selection of participants | Methods: Participants | The Baltimore Longitudinal Study of Aging (BLSA) is a prospective cohort study of community-dwelling participants…Detailed clinical and cognitive evaluations…were conducted every 2 years. Since 2003, participants older than 80 years received yearly assessments.  ADNI is an ongoing, longitudinal study launched in 2003…Details on study design, participant recruitment, study approval, and informed consent procedures have been published previously (11). |
|  |  | (*b*) *Cohort study*—For matched studies, give matching criteria and number of exposed and unexposed  *Case-control study*—For matched studies, give matching criteria and the number of controls per case | Methods: Participants | …metabolomic analyses in serum samples were performed on 207 BLSA (exclusion criteria described below) participants divided into ‘converters’ and ‘non-converters.’ Converters were defined as participants who were cognitively normal at the initial blood draw and developed incident AD based on consensus clinical diagnosis (described below) during follow-up approximately 5 years later. These participants were age- and sex-matched to non-converters… |
| Variables | 7 | Clearly define all outcomes, exposures, predictors, potential confounders, and effect modifiers. Give diagnostic criteria, if applicable | Methods: Determining cognitive status in BLSA and ADNI (Step 2) & Statistical Analysis | In BLSA, cognitive status was considered at consensus diagnosis conferences…  In ADNI, dementia diagnosis was determined based on NINCDS-ADRDA criteria…  Statistical Analysis sections detailing Steps 1 - 4 |
| Data sources/ measurement | 8* | For each variable of interest, give sources of data and details of methods of assessment (measurement). Describe comparability of assessment methods if there is more than one group | Methods: Neuropathological Studies: brain tissue samples in BLSA (Step 1); Determining cognitive status in BLSA and ADNI (Step 2); Blood samples in BLSA and ADNI (Step 2); Brain and Blood metabolomics in BLSA and ADNI (Steps 1 & 2); Cognitive assessments in BLSA (Step 2); Structural MRI measures in ADNI (Step 2); CSF measures of AD pathology in ADNI (Step 2) | See all relevant text in sections identified. |
| Bias | 9 | Describe any efforts to address potential sources of bias | Methods: Statistical analysis: Testing blood metabolite associations with AD endophenotypes in BLSA (sensitivity analyses) | Due to differences in serum sample storage time among converters and non-converters in the BLSA cohort and a greater number of converter samples excluded by the Met-So cutoff, we performed additional sensitivity analyses… |
| Study size | 10 | Explain how the study size was arrived at | Methods: Participants; Blood samples in BLSA and ADNI (Step 2) | ..ADNI…Details on study design, participant recruitment, study approval, and informed consent procedures have been published previously [11].  BLSA…Additional details on sample selection have been published previously [7]. |

| Quantitative variables | 11 | Explain how quantitative variables were handled in the analyses. If applicable, describe which groupings were chosen and why | Methods: Statistical analyses | See all relevant text in sections identified. | | |
| --- | --- | --- | --- | --- | --- | --- |
| Statistical methods | 12 | (*a*) Describe all statistical methods, including those used to control for confounding | Methods: Statistical analyses | See all relevant text in sections identified. | | |
|  |  | (*b*) Describe any methods used to examine subgroups and interactions | Methods: Statistical analyses: Step 2: Testing blood metabolite associations with AD endophenotypes:  Associations with cognitive performance (BLSA) | All models included…the two-way interaction of each predictor with time. The main predictor of interest was the interaction of metabolite concentration with time… | | |
|  |  | (*c*) Explain how missing data were addressed | Methods: Brain and blood metabolomics in BLSA and ADNI (Steps 1 & 2) | BLSA serum samples indicated as less than LOD were not imputed due to minimal missingness; 25/26 metabolites had 0 <LOD values | | |
|  |  | (*d*) *Cohort study*—If applicable, explain how loss to follow-up was addressed  *Case-control study*—If applicable, explain how matching of cases and controls was addressed  *Cross-sectional study*—If applicable, describe analytical methods taking account of sampling strategy | Methods: Participants | These participants were age- and sex-matched to non-converters, and defined as participants who remained cognitively normal over a similar follow-up interval | | |
|  |  | (*e*) Describe any sensitivity analyses | Methods: Statistical analysis: Testing blood metabolite associations with AD endophenotypes in BLSA (sensitivity analyses) | Due to differences in serum sample storage time among converters and non-converters in the BLSA cohort and a greater number of converter samples excluded by the Met-So cutoff, we performed additional sensitivity analyses… |  |  |
| Results | | | | | |  |
| Participants | 13* | (a) Report numbers of individuals at each stage of study—eg numbers potentially eligible, examined for eligibility, confirmed eligible, included in the study, completing follow-up, and analysed | Methods: Fig 1  Results: Table 1 | See Fig1 & Table 1 | | |
|  |  | (b) Give reasons for non-participation at each stage | NA |  | | |
|  |  | (c) Consider use of a flow diagram | Methods: Fig 1 | See Fig 1 | | |
| Descriptive data | 14* | (a) Give characteristics of study participants (eg demographic, clinical, social) and information on exposures and potential confounders | Results: Participants: demographic characteristics | The demographic characteristics… | | |
|  |  | (b) Indicate number of participants with missing data for each variable of interest | NA |  | | |
|  |  | (c) *Cohort study*—Summarise follow-up time (eg, average and total amount) | Results: Step 2: Testing blood metabolite associations with AD endophenotypes: risk of conversion to incident AD in cognitively normal older Adults (BLSA); & Results: Step 2: Testing blood metabolite associations with AD endophenotypes:  Associations with risk of conversion to incident AD (ADNI) | The mean interval between initial blood sampling to the onset of AD (for converters) or follow-up (for non-converters) was 4.27 years…  The mean interval between baseline blood sampling to the onset of AD or follow-up (for individuals who remained MCI) was 2.97 years… | | |
| Outcome data | 15* | *Cohort study*—Report numbers of outcome events or summary measures over time | Results: Table 1 | See Table 1 | | |
|  |  | *Case-control study—*Report numbers in each exposure category, or summary measures of exposure | Results: Table 1 | See Table 1 | | |
|  |  | *Cross-sectional study—*Report numbers of outcome events or summary measures | Results: Table 1 | See Table 1 | | |
| Main results | 16 | (*a*) Give unadjusted estimates and, if applicable, confounder-adjusted estimates and their precision (eg, 95% confidence interval). Make clear which confounders were adjusted for and why they were included | Results | See all relevant text in sections identified. | | |
|  |  | (*b*) Report category boundaries when continuous variables were categorized | NA |  | | |
|  |  | (*c*) If relevant, consider translating estimates of relative risk into absolute risk for a meaningful time period | NA |  | | |

| Other analyses | 17 | Report other analyses done—eg analyses of subgroups and interactions, and sensitivity analyses | Results: Step 2: Testing blood metabolite associations with AD endophenotypes:  Risk of conversion to incident AD in cognitively normal older adults (BLSA); Results: Step 2: exploring associations in blood with AD endophenotypes:  Associations with cognitive performance (BLSA); Results: Step 2: Testing blood metabolite associations with AD endophenotypes:  Associations with AD-like brain atrophy patterns and CSF biomarkers of AD pathology (ADNI) | …metabolites…remained significant in sensitivity analyses conducted in the sub-sample matched on storage time. A summary of the results from the sensitivity analyses is included in … |
| --- | --- | --- | --- | --- |
| Discussion | | | | |
| Key results | 18 | Summarise key results with reference to study objectives | Discussion: paragraph 1 | Our results indicate that distinct metabolites belonging to the sphingolipid and glycerophospholipid classes are related to… |
| Limitations | 19 | Discuss limitations of the study, taking into account sources of potential bias or imprecision. Discuss both direction and magnitude of any potential bias | Discussion: Limitations | Our study has limitations… |
| Interpretation | 20 | Give a cautious overall interpretation of results considering objectives, limitations, multiplicity of analyses, results from similar studies, and other relevant evidence | Discussion: Conclusions | We propose that… |
| Generalisability | 21 | Discuss the generalisability (external validity) of the study results | Discussion: Limitations | …BLSA is a predominantly Caucasian sample… |
| Other information | |  | | |
| Funding | 22 | Give the source of funding and the role of the funders for the present study and, if applicable, for the original study on which the present article is based | Funding | This research was supported in part by… |

*Give information separately for cases and controls in case-control studies and, if applicable, for exposed and unexposed groups in cohort and cross-sectional studies.

**Note:** An Explanation and Elaboration article discusses each checklist item and gives methodological background and published examples of transparent reporting. The STROBE checklist is best used in conjunction with this article (freely available on the Web sites of PLoS Medicine at http://www.plosmedicine.org/, Annals of Internal Medicine at http://www.annals.org/, and Epidemiology at http://www.epidem.com/). Information on the STROBE Initiative is available at www.strobe-statement.org.
