# Supplementary material for: Brain and blood metabolite signatures of pathology and progression in Alzheimer disease: A targeted metabolomics study
Source: PLoS Med. 2018 Jan 25;15(1):e1002482. doi: 10.1371/journal.pmed.1002482 (PMC5784884; doi:10.1371/journal.pmed.1002482)
Supplement: S1 Appendix — (DOCX) [file pmed.1002482.s002.docx]

**S1 Appendix. Description of methods used to generate cognitive domain-specific composite scores**

A summary of each cognitive domain for each visit was calculated by:

1. standardizing the score on each domain specific test on the baseline visit value
2. summing the standardized scores within each domain
3. averaging across the number of domain specific tests included in the summary score

Test included in each domain:

**Memory (verbal)**

CVLT learning

CVLT immediate free recal

**Attention**

Trails Making Test A (this value was log-transformed prior to standardization; sign was also reversed (greater score indicates greater impairment)

Digit Forward (WAIS-R Digits Forward)

**Executive function**

Trails Making Test B (this value was log-transformed prior to standardization; sign was also reversed (greater score indicates greater impairment)

Digit Backward (WAIS-R Digits Backward)

**Language**

Letter Fluency

Semantic Fluency

**Visuo-spatial ability**

Clock Drawing test

Card Rotation test
