# Supplementary material for: Brain and blood metabolite signatures of pathology and progression in Alzheimer disease: A targeted metabolomics study
Source: PLoS Med. 2018 Jan 25;15(1):e1002482. doi: 10.1371/journal.pmed.1002482 (PMC5784884; doi:10.1371/journal.pmed.1002482)
Supplement: S1 Table — ADNI, Alzheimer’s Disease Neuroimaging Initiative. (DOCX) [file pmed.1002482.s003.docx]

**S1 Table. ADNI participating institutions/ study sites**

| **Organization** |
| --- |
| Johns Hopkins University |
| Washington University, St. Louis |
| University of California, Los Angeles |
| University of Pennsylvania |
| Cleveland Clinic Lou Ruvo Center for Brain Health |
| Sunnybrook Health Sciences Centre |
| Parkwood Hospital |
| University of California, San Diego |
| University of Kansas |
| Dent Neurologic Institute |
| McGill University / Jewish General Hospital Memory Clinic |
| Rush University Medical Center |
| Baylor College of Medicine |
| Duke University Medical Center |
| Wein Center for Clinical Research |
| Indiana University |
| St. Joseph’s Health Center – Cognitive Neurology |
| Banner Alzheimer’s Institute |
| New York University Medical Center |
| Mayo Clinic, Jacksonville |
| Mount Sinai School of Medicine |
| University of Michigan, Ann Arbor |
| University of British Columbia, Clinic for AD & Related |
| University of Wisconsin |
| Oregon Health and Science University |
| Northwestern University |
| Boston University |
| Case Western Reserve University |
| Emory University |
| University of Pittsburgh |
| Brigham and Women’s Hospital |
| University of Alabama, Birmingham |
| Medical University of South Carolina |
| University of California, Irvine |
| Howard University |
| University of California, Davis |
| Rhode Island Hospital |
| Mayo Clinic, Rochester |
| Nathan Kline Inst. for Psychiatric Rsch |
| University of Rochester Medical Center |
| University of California, Irvine (BIC) |
| The Weill Cornell Memory Disorders Program |
| Georgetown University |
| University of California, San Francisco |
| Banner Sun Health Research Institute |
| Premiere Research Institute |
| Butler Hospital Memory and Aging Program |
| Dartmouth Medical Center |
| Ohio State University |
| University of Southern California |
| University of Iowa |
| Wake Forest University Health Sciences |
| University of Kentucky |
| University of South Florida, Tampa |
| Columbia University |
| Yale University School of Medicine |
| University of Texas, Southwestern MC |
| Stanford / PAIRE |
| Albany Medical College |
