# Supplementary material for: Brain and blood metabolite signatures of pathology and progression in Alzheimer disease: A targeted metabolomics study
Source: PLoS Med. 2018 Jan 25;15(1):e1002482. doi: 10.1371/journal.pmed.1002482 (PMC5784884; doi:10.1371/journal.pmed.1002482)
Supplement: S2 Table — ITG, inferior temporal gyrus; RF, random forest; SVM, support vector machine. (DOCX) [file pmed.1002482.s004.docx]

**S2 Table. Full ranking from SVM and RF algorithms from the ITG**

| **Random Forest (RF)** | | |  | **Support Vector Machine (SVM)** | | |
| --- | --- | --- | --- | --- | --- | --- |
| **Rank** | **Mean Decrease Accuracy** | **Variables** |  | **Rank** | **Number of CV iterations** | **Variables** |
| 1 | 0.022131 | PC ae C36:0* |  | 1 | 30 | PC aa C40:4* |
| 2 | 0.016173 | SM C16:1* |  | 1 | 30 | PC ae C40:1 |
| 3 | 0.011958 | PC aa C40:4* |  | 1 | 30 | PC ae C42:3* |
| 4 | 0.006392 | SM (OH) C22:1* |  | 1 | 30 | SM (OH) C22:1* |
| 5 | 0.006333 | PC ae C34:0* |  | 1 | 30 | SM (OH) C22:2* |
| 6 | 0.006067 | SM C26:1* |  | 1 | 30 | SM C16:1* |
| 7 | 0.003855 | C3 |  | 7 | 27 | PC ae C36:0* |
| 8 | 0.003767 | SM (OH) C22:2* |  | 8 | 25 | SM C24:1* |
| 9 | 0.003729 | PC ae C42:3* |  | 9 | 23 | SM (OH) C24:1* |
| 10 | 0.003342 | PC aa C40:6* |  | 10 | 22 | Spermidine* |
| 11 | 0.003170 | Serotonin |  | 11 | 6 | PC aa C38:4 |
| 12 | 0.002900 | H1 |  | 12 | 4 | Arg |
| 13 | 0.002893 | lysoPC a C18:0* |  | 12 | 4 | SM C26:1* |
| 14 | 0.002793 | Spermidine* |  | 14 | 2 | lysoPC a C18:0* |
| 15 | 0.002625 | SM C24:1* |  | 14 | 2 | PC aa C40:6* |
| 16 | 0.002452 | SM (OH) C24:1* |  | 16 | 1 | PC ae C34:0* |
| 17 | 0.002036 | lysoPC a C17:0 |  | 16 | 1 | PC ae C34:2 |
| 18 | 0.001907 | SM C18:1 |  | 16 | 1 | PC ae C36:3 |
| 19 | 0.001804 | PC aa C40:5 |  | 16 | 1 | PC ae C36:4 |
| 20 | 0.001799 | SM C16:0 |  | 16 | 1 | SM (OH) C14:1 |
| 21 | 0.001600 | PC ae C40:1 |  |  |  |  |
| 22 | 0.001160 | PC ae C32:1 |  |  |  |  |
| 23 | 0.001093 | PC aa C38:4 |  |  |  |  |
| 24 | 0.001085 | PC ae C34:2 |  |  |  |  |
| 25 | 0.001030 | PC ae C36:3 |  |  |  |  |
| 26 | 0.000837 | SM C24:0 |  |  |  |  |
| 27 | 0.000775 | PC ae C42:1 |  |  |  |  |
| 28 | 0.000701 | PC ae C44:3 |  |  |  |  |
| 29 | 0.000695 | SM (OH) C14:1 |  |  |  |  |
| 30 | 0.000683 | PC ae C38:1 |  |  |  |  |
| 31 | 0.000643 | PC aa C36:4 |  |  |  |  |
| 32 | 0.000596 | Lys |  |  |  |  |
| 33 | 0.000578 | Taurine |  |  |  |  |
| 34 | 0.000545 | lysoPC a C20:4 |  |  |  |  |
| 35 | 0.000477 | PC aa C38:3 |  |  |  |  |
| 36 | 0.000418 | PC ae C36:4 |  |  |  |  |
| 37 | 0.000410 | PC ae C32:2 |  |  |  |  |
| 38 | 0.000399 | PC aa C28:1 |  |  |  |  |
| 39 | 0.000382 | Ala |  |  |  |  |
| 40 | 0.000327 | C2 |  |  |  |  |
| 41 | 0.000298 | Met |  |  |  |  |
| 42 | 0.000297 | SM (OH) C16:1 |  |  |  |  |
| 43 | 0.000273 | PC aa C42:2 |  |  |  |  |
| 44 | 0.000263 | PC ae C36:1 |  |  |  |  |
| 45 | 0.000220 | PC aa C42:1 |  |  |  |  |
| 46 | 0.000212 | C4 |  |  |  |  |
| 47 | 0.000202 | PC aa C30:0 |  |  |  |  |
| 48 | 0.000188 | t4-OH-Pro |  |  |  |  |
| 49 | 0.000176 | Spermine |  |  |  |  |
| 50 | 0.000167 | PC ae C36:2 |  |  |  |  |
| 51 | 0.000154 | C3-DC (C4-OH) |  |  |  |  |
| 52 | 0.000153 | PC aa C32:0 |  |  |  |  |
| 53 | 0.000152 | PC ae C34:3 |  |  |  |  |
| 54 | 0.000146 | PC aa C30:2 |  |  |  |  |
| 55 | 0.000131 | Ile |  |  |  |  |
| 56 | 0.000119 | Asn |  |  |  |  |
| 57 | 0.000118 | PC aa C34:1 |  |  |  |  |
| 58 | 0.000107 | Arg |  |  |  |  |
| 59 | 0.000098 | PC ae C40:6 |  |  |  |  |
| 60 | 0.000090 | Trp |  |  |  |  |
| 61 | 0.000083 | Leu |  |  |  |  |
| 62 | 0.000082 | Asp |  |  |  |  |
| 63 | 0.000078 | Tyr |  |  |  |  |
| 64 | 0.000066 | lysoPC a C16:1 |  |  |  |  |
| 65 | 0.000060 | PC ae C40:2 |  |  |  |  |
| 66 | 0.000052 | PC ae C38:0 |  |  |  |  |
| 67 | 0.000047 | SM C22:3 |  |  |  |  |
| 68 | 0.000047 | Ac-Orn |  |  |  |  |
| 69 | 0.000031 | Phe |  |  |  |  |
| 70 | 0.000027 | PC aa C34:3 |  |  |  |  |
| 71 | 0.000023 | Ser |  |  |  |  |
| 72 | 0.000021 | PC aa C24:0 |  |  |  |  |
| 73 | 0.000019 | PC ae C38:4 |  |  |  |  |
| 74 | 0.000013 | C18 |  |  |  |  |
| 75 | 0.000012 | PC aa C34:2 |  |  |  |  |
| 76 | 0.000006 | PC ae C40:4 |  |  |  |  |
| 77 | 0.000005 | PC aa C32:1 |  |  |  |  |
| 78 | 0.000001 | Histamine |  |  |  |  |
| 79 | 0.000000 | PC aa C42:4 |  |  |  |  |
| 80 | 0.000000 | c4-OH-Pro |  |  |  |  |
| 81 | 0.000000 | Nitro-Tyr |  |  |  |  |
| 82 | 0.000000 | PEA |  |  |  |  |
| 83 | 0.000000 | C3:1 |  |  |  |  |
| 84 | 0.000000 | C3-OH |  |  |  |  |
| 85 | 0.000000 | C4:1 |  |  |  |  |
| 86 | 0.000000 | C5:1 |  |  |  |  |
| 87 | 0.000000 | C5:1-DC |  |  |  |  |
| 88 | 0.000000 | C5-DC (C6-OH) |  |  |  |  |
| 89 | 0.000000 | C5-M-DC |  |  |  |  |
| 90 | 0.000000 | C6 (C4:1-DC) |  |  |  |  |
| 91 | 0.000000 | C7-DC |  |  |  |  |
| 92 | 0.000000 | C8 |  |  |  |  |
| 93 | 0.000000 | C9 |  |  |  |  |
| 94 | 0.000000 | C10 |  |  |  |  |
| 95 | 0.000000 | C10:1 |  |  |  |  |
| 96 | 0.000000 | C10:2 |  |  |  |  |
| 97 | 0.000000 | C12 |  |  |  |  |
| 98 | 0.000000 | C12:1 |  |  |  |  |
| 99 | 0.000000 | C12-DC |  |  |  |  |
| 100 | 0.000000 | C14:1-OH |  |  |  |  |

Note: * indicates consensus metabolites common to both SVM and RF algorithms; top 20 metabolites used to define the brain metabolomic signature of AD
