# Supplementary material for: Brain and blood metabolite signatures of pathology and progression in Alzheimer disease: A targeted metabolomics study
Source: PLoS Med. 2018 Jan 25;15(1):e1002482. doi: 10.1371/journal.pmed.1002482 (PMC5784884; doi:10.1371/journal.pmed.1002482)
Supplement: S3 Table — (DOCX) [file pmed.1002482.s005.docx]

**S3 Table. Brain endophenotype associations: differences by group**

| **metabolite** | **coef** | **stderr** | **ci lower** | **ci upper** | **pval** |
| --- | --- | --- | --- | --- | --- |
| Arg | 1.101913 | 0.6343727 | -.1414347 | 2.345261 | 0.0823851 |
| C3 | 1.883758 | 2.462013 | -2.941699 | 6.709215 | 0.4441945 |
| lysoPC a C17:0 | -3.18474 | 2.117258 | -7.334489 | .9650096 | 0.1325347 |
| lysoPC a C18:0 | -3.890568 | 1.812918 | -7.443821 | -.3373145 | 0.0318709 |
| PC aa C38:4 | -2.69927 | 1.635303 | -5.904404 | .5058651 | 0.0988154 |
| PC aa C40:4 | -5.10478 | 1.773469 | -8.580716 | -1.628843 | 0.0039968 |
| PC aa C40:5 | -2.635859 | 1.627629 | -5.825953 | .5542344 | 0.105351 |
| PC aa C40:6 | -3.135567 | 1.78648 | -6.637003 | .3658688 | 0.079231 |
| PC ae C34:0 | -3.056426 | 1.517866 | -6.03139 | -.0814621 | 0.0440481 |
| PC ae C34:2 | 2.094099 | 1.11009 | -.0816377 | 4.269836 | 0.059238 |
| PC ae C36:0 | -7.299683 | 2.594541 | -12.38489 | -2.214475 | 0.0049009 |
| PC ae C36:3 | 1.707299 | 1.003771 | -.2600569 | 3.674654 | 0.0889647 |
| PC ae C36:4 | 3.333492 | 1.442288 | .5066598 | 6.160325 | 0.0208189 |
| PC ae C40:1 | -5.361838 | 1.940765 | -9.165668 | -1.558008 | 0.0057318 |
| PC ae C42:3 | -5.665544 | 2.469099 | -10.50489 | -.8261987 | 0.0217572 |
| Serotonin | -3.155847 | 2.632442 | -8.31534 | 2.003645 | 0.2305946 |
| SM C16:0 | 3.869462 | 1.393714 | 1.137833 | 6.60109 | 0.005497 |
| SM C16:1 | 3.349897 | 1.397952 | .6099609 | 6.089833 | 0.016562 |
| SM C18:1 | 0.85641 | 0.9752464 | -1.055038 | 2.767858 | 0.3798637 |
| SM C24:1 | 2.410949 | 0.8590716 | .7272 | 4.094699 | 0.0050089 |
| SM C26:1 | 3.139004 | 1.249248 | .6905218 | 5.587485 | 0.0119806 |
| SM (OH) C14:1 | 6.338424 | 2.424836 | 1.585832 | 11.09101 | 0.00895 |
| SM (OH) C22:1 | 5.980365 | 2.348631 | 1.377133 | 10.5836 | 0.0108865 |
| SM (OH) C22:2 | 4.140924 | 1.495118 | 1.210547 | 7.0713 | 0.005612 |
| SM (OH) C24:1 | 9.811586 | 4.905197 | .1975773 | 19.4256 | 0.045474 |
| Spermidine | 4.996655 | 1.604179 | 1.852523 | 8.140788 | 0.0018409 |

Note: all models included covariates age and sex

coef = coefficient; stderr = standard error; pval = p-value; ci = 95% confidence interval
