# Supplementary material for: Brain and blood metabolite signatures of pathology and progression in Alzheimer disease: A targeted metabolomics study
Source: PLoS Med. 2018 Jan 25;15(1):e1002482. doi: 10.1371/journal.pmed.1002482 (PMC5784884; doi:10.1371/journal.pmed.1002482)
Supplement: S4 Table — AD, Alzheimer disease. (DOCX) [file pmed.1002482.s006.docx]

**S4 Table. Brain endophenotype associations: associations with AD pathology**

**CERAD scores**

| **metabolite** | **coef** | **stderr** | **ci lower** | **ci upper** | **pval** |
| --- | --- | --- | --- | --- | --- |
| Arg | 0.0264534 | 0.0150016 | -.0038902 | .0567971 | 0.085672 |
| C3 | 0.0289626 | 0.0138538 | .0009406 | .0569845 | 0.0431312 |
| lysoPC a C17:0 | -0.0324036 | 0.0137053 | -.0601252 | -.0046821 | 0.0231371 |
| lysoPC a C18:0 | -0.0358199 | 0.0139563 | -.0640491 | -.0075907 | 0.0142236 |
| PC aa C38:4 | -0.0209138 | 0.0148719 | -.0509949 | .0091674 | 0.1675641 |
| PC aa C40:4 | -0.0390286 | 0.0137349 | -.06681 | -.0112472 | 0.0071034 |
| PC aa C40:5 | -0.0224676 | 0.0156819 | -.0541872 | .0092521 | 0.1599097 |
| PC aa C40:6 | -0.0301029 | 0.0149641 | -.0603706 | .0001649 | 0.0512001 |
| PC ae C34:0 | -0.0232848 | 0.0161923 | -.0560368 | .0094672 | 0.1584051 |
| PC ae C34:2 | 0.0280297 | 0.0142351 | -.0007636 | .0568229 | 0.0560819 |
| PC ae C36:0 | -0.0368865 | 0.0144 | -.0660132 | -.0077597 | 0.0144005 |
| PC ae C36:3 | 0.0302455 | 0.014097 | .0017316 | .0587594 | 0.038194 |
| PC ae C36:4 | 0.0299006 | 0.014372 | .0008306 | .0589707 | 0.0440977 |
| PC ae C40:1 | -0.0364065 | 0.0138147 | -.0643495 | -.0084636 | 0.0119979 |
| PC ae C42:3 | -0.0429779 | 0.0183277 | -.0800492 | -.0059066 | 0.024212 |
| Serotonin | -0.0192978 | 0.0148117 | -.0492573 | .0106617 | 0.2002583 |
| SM C16:0 | 0.0415372 | 0.0142268 | .0127608 | .0703136 | 0.0057951 |
| SM C16:1 | 0.0378248 | 0.0134717 | .0105757 | .0650739 | 0.0077522 |
| SM C18:1 | 0.0180853 | 0.0142115 | -.0106602 | .0468308 | 0.2107018 |
| SM C24:1 | 0.0411049 | 0.0127676 | .01528 | .0669299 | 0.0025888 |
| SM C26:1 | 0.0357335 | 0.0132108 | .0090121 | .0624548 | 0.0100773 |
| SM (OH) C14:1 | 0.0347897 | 0.0146018 | .0052546 | .0643247 | 0.0221639 |
| SM (OH) C22:1 | 0.0381972 | 0.0133875 | .0111184 | .065276 | 0.0068925 |
| SM (OH) C22:2 | 0.0392432 | 0.0133709 | .012198 | .0662884 | 0.0055663 |
| SM (OH) C24:1 | 0.0265657 | 0.0154098 | -.0046035 | .0577349 | 0.0926365 |
| Spermidine | 0.0409483 | 0.0141485 | .0123303 | .0695663 | 0.0061947 |

**Braak scores**

| **metabolite** | **coef** | **stderr** | **ci lower** | **ci upper** | **pval** |
| --- | --- | --- | --- | --- | --- |
| Arg | 0.0435549 | 0.0186121 | .0059083 | .0812015 | 0.0244872 |
| C3 | 0.0279601 | 0.0180819 | -.008614 | .0645343 | 0.1301089 |
| lysoPC a C17:0 | -0.0358302 | 0.0177829 | -.0717995 | .0001391 | 0.0508493 |
| lysoPC a C18:0 | -0.0261344 | 0.0187787 | -.0641179 | .0118491 | 0.1718993 |
| PC aa C38:4 | -0.0222172 | 0.019109 | -.0608688 | .0164345 | 0.2520359 |
| PC aa C40:4 | -0.0493208 | 0.0175449 | -.0848088 | -.0138328 | 0.0076848 |
| PC aa C40:5 | -0.0297706 | 0.0199533 | -.07013 | .0105888 | 0.1437411 |
| PC aa C40:6 | -0.0183262 | 0.0198297 | -.0584355 | .0217832 | 0.3610767 |
| PC ae C34:0 | -0.034998 | 0.0204319 | -.0763254 | .0063293 | 0.0946687 |
| PC ae C34:2 | 0.003763 | 0.019022 | -.0347126 | .0422386 | 0.8442104 |
| PC ae C36:0 | -0.0527602 | 0.0179574 | -.0890824 | -.016438 | 0.005521 |
| PC ae C36:3 | 0.0050015 | 0.0189889 | -.0334072 | .0434102 | 0.7936367 |
| PC ae C36:4 | 0.0228046 | 0.018967 | -.0155597 | .061169 | 0.2364836 |
| PC ae C40:1 | -0.0506799 | 0.017311 | -.0856947 | -.015665 | 0.0056751 |
| PC ae C42:3 | -0.0247858 | 0.0246446 | -.0746343 | .0250626 | 0.3207455 |
| Serotonin | -0.0329461 | 0.0185572 | -.0704815 | .0045893 | 0.0836388 |
| SM C16:0 | 0.0374367 | 0.0191063 | -.0012094 | .0760828 | 0.0572425 |
| SM C16:1 | 0.0362802 | 0.0179151 | .0000435 | .0725169 | 0.0497386 |
| SM C18:1 | 0.0236758 | 0.0181002 | -.0129353 | .060287 | 0.1985161 |
| SM C24:1 | 0.0213878 | 0.0179924 | -.0150054 | .0577809 | 0.2417426 |
| SM C26:1 | 0.0139078 | 0.0182212 | -.0229479 | .0507636 | 0.4498896 |
| SM (OH) C14:1 | 0.0259469 | 0.0194893 | -.0134738 | .0653677 | 0.1908059 |
| SM (OH) C22:1 | 0.0106677 | 0.0186892 | -.0271348 | .0484702 | 0.5714148 |
| SM (OH) C22:2 | 0.0208505 | 0.0185395 | -.0166491 | .0583501 | 0.2676098 |
| SM (OH) C24:1 | 0.0345717 | 0.0196172 | -.0051078 | .0742512 | 0.0858525 |
| Spermidine | 0.0256549 | 0.0194544 | -.0136952 | .0650051 | 0.1949531 |

Note: all models included covariates age and sex

coef = coefficient; stderr = standard error; pval = p-value; ci = 95% confidence interval
