# Supplementary material for: Brain and blood metabolite signatures of pathology and progression in Alzheimer disease: A targeted metabolomics study
Source: PLoS Med. 2018 Jan 25;15(1):e1002482. doi: 10.1371/journal.pmed.1002482 (PMC5784884; doi:10.1371/journal.pmed.1002482)
Supplement: S5 Table — AD, Alzheimer disease; BLSA, Baltimore Longitudinal Study of Aging. (DOCX) [file pmed.1002482.s007.docx]

**S5 Table. Blood endophenotype associations: risk of progression to incident AD in cognitively normal older individuals (BLSA)**

| **metabolite** | **coef** | **stderr** | **ci lower** | **ci upper** | **pval** |
| --- | --- | --- | --- | --- | --- |
| Arg | .5709435 | .2240472 | .2645869 | 1.232021 | 0.1532222 |
| C3 | .0897175 | .1239936 | .0059769 | 1.346716 | 0.0810555 |
| lysoPC a C17:0 | .6481476 | .3297502 | .2391214 | 1.756829 | 0.3940246 |
| lysoPC a C18:0 | .7156401 | .2747499 | .3372094 | 1.518762 | 0.3834955 |
| PC aa C38:4 | .2532355 | .117757 | .1017905 | .6300018 | 0.0031411 |
| PC aa C40:4 | 1.01543 | .5219437 | .3707848 | 2.780854 | 0.9762347 |
| PC aa C40:5 | .9062348 | .4299908 | .3575708 | 2.29678 | 0.8356158 |
| PC aa C40:6 | .7698249 | .2343601 | .4238949 | 1.39806 | 0.3901872 |
| PC ae C34:0 | 4.27222 | 3.307418 | .9368718 | 19.48171 | 0.0606902 |
| PC ae C34:2 | 3.054504 | 1.441946 | 1.210911 | 7.704939 | 0.0180114 |
| PC ae C36:0 | 1.933932 | 2.000543 | .2546371 | 14.68794 | 0.5237396 |
| PC ae C36:3 | 2.938174 | 1.677273 | .959764 | 8.994783 | 0.0590221 |
| PC ae C36:4 | .9700565 | .5984486 | .289516 | 3.250285 | 0.9606973 |
| PC ae C40:1 | .3266478 | .2541833 | .0710741 | 1.501233 | 0.1504758 |
| PC ae C42:3 | .5152974 | .5437465 | .0651422 | 4.076181 | 0.5297932 |
| Serotonin | 1.117527 | .6924377 | .3317723 | 3.76423 | 0.8576747 |
| SM C16:0 | 4.430268 | 2.160129 | 1.703717 | 11.52026 | 0.0022675 |
| SM C16:1 | 3.455207 | 1.451883 | 1.516344 | 7.873181 | 0.0031705 |
| SM C18:1 | 2.254542 | .882247 | 1.047047 | 4.854565 | 0.0377596 |
| SM C24:1 | 1.852773 | .9690652 | .6646818 | 5.164526 | 0.2383793 |
| SM C26:1 | 2.965045 | 4.448389 | .1566727 | 56.11376 | 0.4687823 |
| SM (OH) C14:1 | 3.539119 | 1.709591 | 1.373149 | 9.121636 | 0.008884 |
| SM (OH) C22:1 | 1.55834 | .9319698 | .4826124 | 5.031828 | 0.4582247 |
| SM (OH) C22:2 | 1.51043 | .826884 | .5165479 | 4.416624 | 0.4512694 |
| SM (OH) C24:1 | 3.544948 | 3.072874 | .648278 | 19.38467 | 0.1443063 |
| Spermidine | 2.130793 | 5.506967 | .0134474 | 337.6332 | 0.7697456 |

Note: all models included covariates age and sex; individuals who remained normal (non-converters) were censored at the last follow-up visit

coef = coefficient; stderr = standard error; pval = p-value; ci = 95% confidence interval
