# Supplementary material for: Brain and blood metabolite signatures of pathology and progression in Alzheimer disease: A targeted metabolomics study
Source: PLoS Med. 2018 Jan 25;15(1):e1002482. doi: 10.1371/journal.pmed.1002482 (PMC5784884; doi:10.1371/journal.pmed.1002482)
Supplement: S6 Table — Blood endophenotype associations: risk of progression to incident AD in cognitively normal older individuals (BLSA). AD, Alzheimer disease; BLSA, Baltimore Longitudinal Study of Aging. (DOCX) [file pmed.1002482.s008.docx]

**S6 Table. Sensitivity analyses in sub-sample matched on storage time: Blood endophenotype associations: risk of progression to incident AD in cognitively normal older individuals (BLSA)**

| **metabolite** | **coef** | **stderr** | **ci lower** | **ci upper** | **pval** |
| --- | --- | --- | --- | --- | --- |
| Arg | .4776549 | .2102435 | .2015818 | 1.13182 | .0932221 |
| C3 | .084291 | .1316587 | .0039468 | 1.800194 | .1132893 |
| lysoPC a C17:0 | .5302922 | .2865762 | .1838721 | 1.529378 | .2404814 |
| lysoPC a C18:0 | .6243978 | .2610926 | .2751253 | 1.417073 | .2600344 |
| PC aa C38:4 | .3368922 | .1816639 | .1170829 | .9693672 | .0436266* |
| PC aa C40:4 | .7506933 | .4240396 | .248113 | 2.271306 | .6116928 |
| PC aa C40:5 | 1.015084 | .5194322 | .3723322 | 2.767409 | .9766594 |
| PC aa C40:6 | 1.025365 | .4156001 | .4633084 | 2.269273 | .9507224 |
| PC ae C34:0 | 4.116708 | 3.396736 | .8169873 | 20.74363 | .0863473 |
| PC ae C34:2 | 3.433389 | 1.779236 | 1.243418 | 9.480449 | .017295* |
| PC ae C36:0 | 1.608395 | 4.174822 | .3208095 | 37.0236 | .307051 |
| PC ae C36:3 | 2.479389 | 1.608395 | .6952886 | 8.841466 | .1615947 |
| PC ae C36:4 | 1.045874 | .772909 | .245717 | 4.451679 | .951603 |
| PC ae C40:1 | .4396291 | .4377737 | .0624421 | 3.095249 | .4091973 |
| PC ae C42:3 | 1.317134 | 1.675685 | .1088213 | 15.94211 | .8285843 |
| Serotonin | .6045175 | .4147246 | .157561 | 2.319365 | .4631535 |
| SM C16:0 | 4.32194 | 2.219957 | 1.579285 | 11.82761 | .0043771* |
| SM C16:1 | 3.958175 | 1.837749 | 1.593268 | 9.83334 | .0030447* |
| SM C18:1 | 2.819817 | 1.358496 | 1.096831 | 7.249401 | .0314123* |
| SM C24:1 | 3.671577 | 2.330109 | 1.058402 | 12.73663 | .0404228 |
| SM C26:1 | 8.057115 | 14.41445 | .2417417 | 268.5391 | .2434918 |
| SM (OH) C14:1 | 3.299509 | 1.680558 | 1.215912 | 8.953579 | .0190891* |
| SM (OH) C22:1 | 1.690369 | 1.049963 | .5003371 | 5.710846 | .3980387 |
| SM (OH) C22:2 | 3.058811 | 1.985183 | .8572722 | 10.91406 | .0849468 |
| SM (OH) C24:1 | 3.150693 | 2.891053 | .5216144 | 19.03104 | .2110483 |
| Spermidine | .8862602 | 2.535803 | .0032514 | 241.5754 | .9663392 |

Note: all models included covariates age and sex; individuals who remained normal (non-converters) were censored at the last follow-up visit

coef = coefficient; stderr = standard error; pval = p-value; ci = 95% confidence interval

*****Metabolites showing significant associations in the original dataset (i.e. before matching on sample storage duration - S6 Table)
