# Supplementary material for: Brain and blood metabolite signatures of pathology and progression in Alzheimer disease: A targeted metabolomics study
Source: PLoS Med. 2018 Jan 25;15(1):e1002482. doi: 10.1371/journal.pmed.1002482 (PMC5784884; doi:10.1371/journal.pmed.1002482)
Supplement: S7 Table — BLSA, Baltimore Longitudinal Study of Aging. (DOCX) [file pmed.1002482.s009.docx]

**S7 Table.** **Blood endophenotype associations: cognitive performance (BLSA)**

**Memory**

| **metabolite** | **coef** | **stderr** | **ci lower** | **ci upper** | **pval** |
| --- | --- | --- | --- | --- | --- |
| - |  |  |  |  |  |

**Attention**

| **metabolite** | **coef** | **stderr** | **ci lower** | **ci upper** | **pval** |
| --- | --- | --- | --- | --- | --- |
| PC aa C40:6 | -0.1223027 | 0.0471654 | -0.2147452 | -0.0298602 | 0.0095126 |
| SM C18:1 | -0.1719903 | 0.0682364 | -0.3057312 | -0.0382495 | 0.0117185 |

**Executive Function**

| **metabolite** | **coef** | **stderr** | **ci lower** | **ci upper** | **pval** |
| --- | --- | --- | --- | --- | --- |
| - |  |  |  |  |  |

**Language**

| **Metabolite** | **coef** | **stderr** | **ci lower** | **ci upper** | **pval** |
| --- | --- | --- | --- | --- | --- |
| Arg | -0.1418625 | 0.0655939 | -0.2704242 | -0.0133009 | 0.0305611 |
| lysoPC a C18:0 | -0.1495686 | 0.0594351 | -0.2660592 | -0.0330779 | 0.0118526 |
| PC ae C40:1 | -0.2510892 | 0.1228489 | -0.4918685 | -0.0103098 | 0.0409647 |
| SM C26:1 | -0.5334591 | 0.2693737 | -1.061422 | -0.0054965 | 0.0476621 |

**Visuospatial ability**

| **metabolite** | **coef** | **stderr** | **ci lower** | **ci upper** | **pval** |
| --- | --- | --- | --- | --- | --- |
| Arg | 0.1982468 | 0.0978242 | 0.006515 | 0.3899787 | 0.0427071 |
| Spermidine | 1.220205 | 0.574743 | 0.0937289 | 2.34668 | 0.0337502 |

Note: all models included covariates age and sex

coef = coefficient; stderr = standard error; pval = p-value; ci = 95% confidence interval
