# Supplementary material for: Brain and blood metabolite signatures of pathology and progression in Alzheimer disease: A targeted metabolomics study
Source: PLoS Med. 2018 Jan 25;15(1):e1002482. doi: 10.1371/journal.pmed.1002482 (PMC5784884; doi:10.1371/journal.pmed.1002482)
Supplement: S8 Table — BLSA, Baltimore Longitudinal Study of Aging. (DOCX) [file pmed.1002482.s010.docx]

**S8 Table.** **Sensitivity analyses in sub-sample matched on storage time: Blood endophenotype associations: cognitive performance (BLSA)**

**Memory**

| **metabolite** | **coef** | **stderr** | **ci lower** | **ci upper** | **pval** |
| --- | --- | --- | --- | --- | --- |
| Spermidine | -1.487965 | 0.697182 | -2.854417 | -0.1215133 | 0.0328218 |

**Attention**

| **Metabolite** | **coef** | **stderr** | **ci lower** | **ci upper** | **pval** |
| --- | --- | --- | --- | --- | --- |
| PC aa C40:6 | -0.1318884 | 0.0620411 | -0.2534868 | -0.01029 | 0.0335182* |
| SM C26:1 | -0.5653301 | 0.2865502 | -1.126958 | -0.0037021 | 0.0485088* |

**Executive Function**

| **metabolite** | **coef** | **stderr** | **ci lower** | **ci upper** | **pval** |
| --- | --- | --- | --- | --- | --- |
| - |  |  |  |  |  |

**Language**

| **Metabolite** | **coef** | **stderr** | **ci lower** | **ci upper** | **pval** |
| --- | --- | --- | --- | --- | --- |
| Arg | -0.1901535 | 0.0743502 | -0.3358772 | -0.0444298 | 0.0105416* |
| lysoPC a C17:0 | -0.1890103 | 0.0896377 | -0.3646969 | -0.0133237 | 0.0349788 |
| lysoPC a C18:0 | -0.1736529 | 0.0618184 | -0.2948148 | -0.052491 | 0.0049683* |
| PC ae C34:0 | -0.2984266 | 0.1369472 | -0.5668383 | -0.030015 | 0.0293216 |
| SM C26:1 | -0.6001887 | 0.296669 | -1.181649 | -0.0187282 | 0.0430636* |

**Visuospatial ability**

| **metabolite** | **coef** | **stderr** | **ci lower** | **ci upper** | **pval** |
| --- | --- | --- | --- | --- | --- |
| Arg | 0.2361264 | 0.1083943 | 0.0236775 | 0.4485752 | 0.0293761* |

Note: all models included covariates age and sex

coef = coefficient; stderr = standard error; pval = p-value; ci = 95% confidence interval

*****Metabolites showing significant associations in the original dataset (i.e. before matching on sample storage duration – S7 Table)
