# Supplementary material for: Brain and blood metabolite signatures of pathology and progression in Alzheimer disease: A targeted metabolomics study
Source: PLoS Med. 2018 Jan 25;15(1):e1002482. doi: 10.1371/journal.pmed.1002482 (PMC5784884; doi:10.1371/journal.pmed.1002482)
Supplement: S9 Table — AD, Alzheimer disease; ADNI, Alzheimer’s Disease Neuroimaging Initiative; CSF, cerebrospinal fluid. (DOCX) [file pmed.1002482.s011.docx]

**S9 Table. Blood endophenotype associations: AD-like brain atrophy patterns (ADNI)**

| **metabolite** | **coef** | **stderr** | **ci lower** | **ci upper** | **pval** |
| --- | --- | --- | --- | --- | --- |
| Arg | -.2929505 | .257261 | -.7979751 | .212074 | .2551742 |
| C3 | -2.45864 | .6636165 | -3.761374 | -1.155907 | .0002268 |
| lysoPC a C17:0 | .0342361 | .239817 | -.4365444 | .5050166 | .886518 |
| lysoPC a C18:0 | .1427564 | .2064159 | -.2624549 | .5479678 | .4894016 |
| PC aa C38:4 | .2146666 | .23486 | -.2463829 | .6757162 | .3609957 |
| PC aa C40:4 | .1995703 | .2102299 | -.2131283 | .6122688 | .3427714 |
| PC aa C40:5 | .1187087 | .2004075 | -.2747077 | .512125 | .5538011 |
| PC aa C40:6 | -.3228115 | .1498322 | -.6169444 | -.0286787 | .0315147 |
| PC ae C34:0 | .2549883 | .2915869 | -.3174208 | .8273974 | .3821298 |
| PC ae C34:2 | .2154671 | .1933124 | -.1640209 | .5949552 | .2653707 |
| PC ae C36:0 | .4058873 | .3639913 | -.3086576 | 1.120432 | .2651585 |
| PC ae C36:3 | .1444957 | .2053842 | -.2586904 | .5476817 | .4819353 |
| PC ae C36:4 | -.1259803 | .2000497 | -.5186942 | .2667336 | .5290501 |
| PC ae C40:1 | -.1083563 | .3370493 | -.7700118 | .5532992 | .747931 |
| PC ae C42:3 | .3895465 | .4310171 | -.4565755 | 1.235668 | .3663954 |
| Serotonin | -.7099598 | .198393 | -1.099422 | -.320498 | .0003674 |
| SM (OH) C14:1 | .2490647 | .2190683 | -.1809844 | .6791139 | .2559261 |
| SM (OH) C22:1 | .012593 | .2170298 | -.4134542 | .4386402 | .9537445 |
| SM (OH) C22:2 | .2069187 | .2213058 | -.2275228 | .6413602 | .3500897 |
| SM (OH) C24:1 | -.2657606 | .2944678 | -.843825 | .3123039 | .3670704 |
| SM C16:0 | .593261 | .227377 | .1469013 | 1.039621 | .0092547 |
| SM C16:1 | .4196734 | .2465568 | -.0643378 | .9036846 | .0891376 |
| SM C18:1 | .4658887 | .2023309 | .0686965 | .8630809 | .0215696 |
| SM C24:1 | .217659 | .2188233 | -.2119091 | .647227 | .3202081 |
| SM C26:1 | .5126536 | .4608996 | -.3921301 | 1.417437 | .2663646 |
| Spermidine | .3660288 | .8967737 | -1.394412 | 2.126469 | .6832696 |

Note: all models included covariates age and sex

coef = coefficient; stderr = standard error; pval = p-value; ci = 95% confidence interval
