# Supplementary material for: Brain and blood metabolite signatures of pathology and progression in Alzheimer disease: A targeted metabolomics study
Source: PLoS Med. 2018 Jan 25;15(1):e1002482. doi: 10.1371/journal.pmed.1002482 (PMC5784884; doi:10.1371/journal.pmed.1002482)
Supplement: S10 Table — AD, Alzheimer disease; ADNI, Alzheimer’s Disease Neuroimaging Initiative; CSF, cerebrospinal fluid. (DOCX) [file pmed.1002482.s012.docx]

**S10 Table. Blood endophenotype associations: CSF biomarkers of AD pathology (ADNI)**

**t-tau**

| **metabolite** | **coef** | **stderr** | **ci lower** | **ci upper** | **pval** |
| --- | --- | --- | --- | --- | --- |
| PC aa C38:4 | 0.2785333 | 0.1236398 | .0354416 | .521625 | 0.0248339 |
| PC ae C34:0 | 0.358039 | 0.1552326 | .0528317 | .6632463 | 0.0216135 |
| SM (OH) C14:1 | 0.3457978 | 0.1206887 | .1085083 | .5830873 | 0.0043953 |
| SM (OH) C22:1 | 0.2672847 | 0.121657 | .0280914 | .506478 | 0.0286117 |
| SM (OH) C22:2 | 0.2699029 | 0.1162943 | .0412533 | .4985526 | 0.0208147 |
| SM C16:0 | 0.3474697 | 0.12446 | .1027653 | .5921741 | 0.0055018 |
| SM C16:1 | 0.3391437 | 0.1311859 | .0812152 | .5970721 | 0.0100978 |
| SM C18:1 | 0.3806137 | 0.1102869 | .1637754 | .597452 | 0.00062 |

**p-tau**

| **metabolite** | **coef** | **stderr** | **ci lower** | **ci upper** | **pval** |
| --- | --- | --- | --- | --- | --- |
| PC aa C38:4 | 0.2507026 | 0.1236514 | .0075977 | .4938074 | 0.0432897 |
| PC ae C34:0 | 0.4228823 | 0.155191 | .1177691 | .7279955 | 0.0067206 |
| SM (OH) C14:1 | 0.4158154 | 0.1204581 | .1789888 | .652642 | 0.0006173 |
| SM (OH) C22:1 | 0.2923577 | 0.1215118 | .0534594 | .5312561 | 0.0165915 |
| SM (OH) C22:2 | 0.2995711 | 0.1163398 | .0708413 | .5283009 | 0.0103918 |
| SM (OH) C24:1 | 0.372436 | 0.1594876 | .0588755 | .6859965 | 0.0200379 |
| SM C16:0 | 0.3305649 | 0.124543 | .0857072 | .5754225 | 0.0082736 |
| SM C16:1 | 0.3496278 | 0.131345 | .091397 | .6078585 | 0.0080905 |
| SM C18:1 | 0.3106996 | 0.1106741 | .0931089 | .5282903 | 0.0052454 |
| SM C26:1 | 0.5740063 | 0.2492435 | .0839813 | 1.064031 | 0.0218034 |

**Aβ1-42**

| **metabolite** | **coef** | **stderr** | **ci lower** | **ci upper** | **pval** |
| --- | --- | --- | --- | --- | --- |
| C3 | 0.4927783 | 0.2351658 | .030427 | .9551295 | 0.0367752 |
| SM (OH) C14:1 | -0.1789616 | 0.0782791 | -.3328634 | -.0250599 | 0.022778 |
| SM C16:0 | -0.1693469 | 0.0806385 | -.3278875 | -.0108064 | 0.036364 |
| Serotonin | 0.1984766 | 0.0674346 | .0658958 | .3310573 | 0.0034421 |

Note: all models included covariates age and sex

coef = coefficient; stderr = standard error; pval = p-value; ci = 95% confidence interval
