# Supplementary material for: Brain and blood metabolite signatures of pathology and progression in Alzheimer disease: A targeted metabolomics study
Source: PLoS Med. 2018 Jan 25;15(1):e1002482. doi: 10.1371/journal.pmed.1002482 (PMC5784884; doi:10.1371/journal.pmed.1002482)
Supplement: S11 Table — AD, Alzheimer disease; ADNI, Alzheimer’s Disease Neuroimaging Initiative; MCI, mild cognitive impairment. (DOCX) [file pmed.1002482.s013.docx]

**S11 Table. Blood endophenotype associations: risk of progression to incident AD in MCI individuals (ADNI)**

| **metabolite** | **coef** | **stderr** | **ci lower** | **ci upper** | **pval** |
| --- | --- | --- | --- | --- | --- |
| Arg | .6037805 | .2300671 | .2861104 | 1.274162 | 0.1854666 |
| C3 | .1620079 | .1858833 | .0170959 | 1.535256 | 0.1126645 |
| lysoPC a C17:0 | 1.099539 | .3928377 | .5458808 | 2.214745 | 0.7905495 |
| lysoPC a C18:0 | .9040152 | .2741608 | .498921 | 1.638022 | 0.7393331 |
| PC aa C38:4 | 2.375218 | .8385966 | 1.188988 | 4.744926 | 0.0142755 |
| PC aa C40:4 | 1.674515 | .4887355 | .9450424 | 2.967062 | 0.0773463 |
| PC aa C40:5 | 1.753102 | .5252242 | .974518 | 3.153732 | 0.060957 |
| PC aa C40:6 | .9187124 | .2019222 | .5971666 | 1.413395 | 0.6996857 |
| PC ae C34:0 | 1.843792 | .8095346 | .7798006 | 4.359535 | 0.1634725 |
| PC ae C34:2 | .8925078 | .2627672 | .5011941 | 1.589345 | 0.6993054 |
| PC ae C36:0 | 1.24506 | .6708193 | .4330878 | 3.579354 | 0.6841465 |
| PC ae C36:3 | .9024742 | .2862011 | .4847231 | 1.680258 | 0.7462598 |
| PC ae C36:4 | .9259596 | .2728991 | .5196676 | 1.649903 | 0.7940857 |
| PC ae C40:1 | .9430537 | .4758495 | .3507794 | 2.535355 | 0.907495 |
| PC ae C42:3 | 1.319058 | .8688567 | .3627287 | 4.796737 | 0.6741903 |
| Serotonin | 1.369671 | .4389797 | .7308131 | 2.567004 | 0.3263461 |
| SM C16:0 | 1.489343 | .5022603 | .7690216 | 2.884368 | 0.2375336 |
| SM C16:1 | 1.910188 | .7283725 | .9047006 | 4.033176 | 0.0896375 |
| SM C18:1 | 2.351555 | .7407403 | 1.268313 | 4.359973 | 0.006637 |
| SM C24:1 | 1.674971 | .5328514 | .9491514 | 5.284263 | 0.1049409 |
| SM C26:1 | 2.029173 | 1.290812 | .5832393 | 7.059785 | 0.2659657 |
| SM (OH) C14:1 | 1.573355 | .5108415 | .8326374 | 2.973016 | 0.1627585 |
| SM (OH) C22:1 | 1.505622 | .4904869 | .795096 | 2.851097 | 0.2090735 |
| SM (OH) C22:2 | 1.623991 | .5559475 | .8302103 | 3.17672 | 0.1566547 |
| SM (OH) C24:1 | 2.239546 | .9809164 | .8978796 | 3.124614 | 0.0656486 |
| Spermidine | .8012972 | 1.12288 | .0514022 | 12.49125 | 0.8743929 |

Note: all models included covariates age and sex; individuals who remained MCI were censored at the last follow-up visit

coef = coefficient; stderr = standard error; pval = p-value; ci = 95% confidence interval
