# Supplementary material for: Brain and blood metabolite signatures of pathology and progression in Alzheimer disease: A targeted metabolomics study
Source: PLoS Med. 2018 Jan 25;15(1):e1002482. doi: 10.1371/journal.pmed.1002482 (PMC5784884; doi:10.1371/journal.pmed.1002482)
Supplement: S12 Table — AD, Alzheimer disease. (DOCX) [file pmed.1002482.s014.docx]

**S12 Table. P-values for all brain and blood-specific AD endophenotypes included in Figure 4**

| **Metabolite** | **AD-ASY-CN** | **CERAD** | **Braak** | **SPARE-AD** | **A Beta** | **t-tau** | **p-tau** | **Risk: MCI-AD** | **Risk: CN-AD** | **Memory** | **Language** | **Visuospatial** | **Attention** | **Exec fx** | **EASEAD** |
| --- | --- | --- | --- | --- | --- | --- | --- | --- | --- | --- | --- | --- | --- | --- | --- |
| SM C16:0 | .005497 | .0057951 | .0572425 | .0092547 | .036364 | .0055018 | .0082736 | .2375336 | .0022677 | .939869 | .3939669 | .7660454 | .2386464 | .5678734 | 7 |
| SM C18:1 | .3798637 | .2107018 | .1985161 | .0215696 | .1104083 | .00062 | .0052454 | .006637 | .0377602 | .9162469 | .0827978 | .6247497 | .0117185 | .6961749 | 6 |
| SM C16:1 | .016562 | .0077522 | .0497386 | .0891376 | .1542054 | .0100978 | .0080905 | .0896375 | .0031707 | .9165261 | .2293332 | .8064792 | .2028824 | .4513896 | 6 |
| SM (OH) C14:1 | .00895 | .0221639 | .1908059 | .2559261 | .022778 | .0043953 | .0006173 | .1627585 | .0088856 | .8690444 | .6960021 | .4476385 | .6334401 | .98595 | 6 |
| SM C26:1 | .0119806 | .0100773 | .4498896 | .2663646 | .252343 | .0535474 | .0218034 | .2659657 | .4687826 | .8242786 | .0476621 | .5894005 | .2637807 | .3351556 | 4 |
| SM (OH) C22:2 | .005612 | .0055663 | .2676098 | .3500897 | .1168478 | .0208147 | .0103918 | .1566547 | .4512693 | .1112222 | .7330758 | .8778779 | .503178 | .5602561 | 4 |
| SM (OH) C22:1 | .0108865 | .0068925 | .5714148 | .9537445 | .1850196 | .0286117 | .0165915 | .2090735 | .4582235 | .4943246 | .1981034 | .7414488 | .6271206 | .6996439 | 4 |
| PC ae C40:1 | .0057318 | .0119979 | .0056751 | .747931 | .2137569 | .0730496 | .3355083 | .907495 | .1504767 | .5479468 | .0409647 | .1528269 | .060671 | .3525568 | 4 |
| PC aa C38:4 | .0988154 | .1675641 | .2520359 | .3609957 | .3637815 | .0248339 | .0432897 | .0142755 | .0031413 | .6142179 | .6614042 | .6022653 | .1507145 | .8409258 | 4 |
| PC ae C36:0 | .0049009 | .0144005 | .005521 | .2651585 | .2954025 | .2361209 | .2273254 | .6841465 | .5237375 | .6889758 | .1364394 | .5420429 | .0545646 | .1221705 | 3 |
| PC ae C34:0 | .0440481 | .1584051 | .0946687 | .3821298 | .2108823 | .0216135 | .0067206 | .1634725 | .0606919 | .9434205 | .0965219 | .6018404 | .7069029 | .4409404 | 3 |
| PC aa C40:4 | .0039968 | .0071034 | .0076848 | .3427714 | .4863949 | .4198788 | .4859705 | .0773463 | .9762347 | .9347603 | .5618659 | .7376142 | .9755365 | .9828357 | 3 |
| C3 | .4441945 | .0431312 | .1301089 | .0002268 | .0367752 | .2888157 | .1530835 | .1126645 | .0810578 | .2439509 | .5401747 | .7147278 | .1974714 | .0842114 | 3 |
| lysoPC a C18:0 | .0318709 | .0142236 | .1718993 | .4894016 | .1493457 | .1789643 | .219912 | .7393331 | .3834955 | .1952575 | .0118526 | .1924446 | .09868 | .5267406 | 3 |
| Spermidine | .0018409 | .0061947 | .1949531 | .6832696 | .3392596 | .5469232 | .8660314 | .8743929 | .7697456 | .1500387 | .3375619 | .0337502 | .4051005 | .8598805 | 3 |
| SM (OH) C24:1 | .045474 | .0926365 | .0858525 | .3670704 | .3573725 | .078138 | .0200379 | .0656486 | .1443062 | .6989764 | .287315 | .4460312 | .8768055 | .6545058 | 2 |
| SM C24:1 | .0050089 | .0025888 | .2417426 | .3202081 | .4586234 | .2551502 | .1064531 | .1049409 | .2383793 | .4781859 | .9528276 | .3656681 | .0909951 | .184817 | 2 |
| PC ae C36:4 | .0208189 | .0440977 | .2364836 | .5290501 | .7100291 | .4308324 | .5655225 | .7940857 | .9606973 | .382136 | .5496569 | .8670484 | .2701713 | .2475574 | 2 |
| PC ae C42:3 | .0217572 | .024212 | .3207455 | .3663954 | .074711 | .1053158 | .2751024 | .6741903 | .5297931 | .1716313 | .088467 | .2264684 | .2116118 | .1805146 | 2 |
| PC aa C40:6 | .079231 | .0512001 | .3610767 | .0315147 | .4025719 | .5643542 | .4674217 | .6996857 | .3901879 | .956551 | .2170281 | .0835394 | .0095126 | .284438 | 2 |
| Arg | .0823851 | .085672 | .0244872 | .2551742 | .6415608 | .7105517 | .5816172 | .1854666 | .1532218 | .1961152 | .0305611 | .0427071 | .2763251 | .6346112 | 2 |
| Serotonin | .2305946 | .2002583 | .0836388 | .0003674 | .0034421 | .2036715 | .3270973 | .3263461 | .8576753 | .6779586 | .4665209 | .3356874 | .2716917 | .615286 | 2 |
| PC ae C34:2 | .059238 | .0560819 | .8442104 | .2653707 | .1227462 | .055406 | .0733009 | .6993054 | .0180129 | .9559034 | .7072402 | .1448226 | .6919867 | .6396387 | 1 |
| PC ae C36:3 | .0889647 | .038194 | .7936367 | .4819353 | .1438533 | .0991377 | .2320458 | .7462598 | .0590228 | .8040131 | .5534921 | .1904369 | .6904622 | .6083636 | 1 |
| lysoPC a C17:0 | .1325347 | .0231371 | .0508493 | .886518 | .1296147 | .0535568 | .0623291 | .7905495 | .3940231 | .9294038 | .1292699 | .5481932 | .2462647 | .6135386 | 1 |
| PC aa C40:5 | .105351 | .1599097 | .1437411 | .5538011 | .3609647 | .1130895 | .055678 | .060957 | .8356158 | .7991161 | .4514883 | .915031 | .3653652 | .5003752 | 0 |

Note: cognitive tests (highlighted in gray) were collapsed in Figure 4 into “any significant” association across all tests
